# Umbilical cord extracts improve osteoporotic abnormalities of bone marrow-derived mesenchymal stem cells and promote their therapeutic effects on ovariectomised rats

**DOI:** 10.1038/s41598-018-19516-6

**Published:** 2018-01-18

**Authors:** Akira Saito, Kanna Nagaishi, Kousuke Iba, Yuka Mizue, Takako Chikenji, Miho Otani, Masako Nakano, Kazusa Oyama, Toshihiko Yamashita, Mineko Fujimiya

**Affiliations:** 1 Department of Orthopaedic Surgery, Sapporo Medical University, Sapporo, Japan; 2 Second Department of Anatomy, Sapporo Medical University, Sapporo, Japan; 3 Department of Diabetic Cellular Therapeutics, Sapporo Medical University, Sapporo, Japan

## Abstract

Bone marrow-derived mesenchymal stem cells (BM-MSCs) are the most valuable source of autologous cells for transplantation and tissue regeneration to treat osteoporosis. Although BM-MSCs are the primary cells responsible for maintaining bone metabolism and homeostasis, their regenerative ability may be attenuated in postmenopausal osteoporosis patients. Therefore, we first examined potential abnormalities of BM-MSCs in an oestrogen-deficient rat model constructed by ovariectomy (OVX-MSCs). Cell proliferation, mobilisation, and regulation of osteoclasts were downregulated in OVX-MSCs. Moreover, therapeutic effects of OVX-MSCs were decreased in OVX rats. Accordingly, we developed a new activator for BM-MSCs using human umbilical cord extracts, Wharton’s jelly extract supernatant (WJS), which improved cell proliferation, mobilisation and suppressive effects on activated osteoclasts in OVX-MSCs. Bone volume, RANK and TRACP expression of osteoclasts, as well as proinflammatory cytokine expression in bone tissues, were ameliorated by OVX-MSCs activated with WJS (OVX-MSCs-WJ) in OVX rats. Fusion and bone resorption activity of osteoclasts were suppressed in macrophage-induced and primary mouse bone marrow cell-induced osteoclasts via suppression of osteoclast-specific genes, such as *Nfatc1*, *Clcn7*, *Atp6i* and *Dc-stamp*, by co-culture with OVX-MSCs-WJ *in vitro*. In this study, we developed a new activator, WJS, which improved the functional abnormalities and therapeutic effects of BM-MSCs on postmenopausal osteoporosis.

## Introduction

Postmenopausal osteoporosis, the most frequent form of osteoporosis, is caused by a reduction of oestrogen secretion accompanied by a decrease in ovarian function. As oestrogen maintains bone density by suppressing bone resorption^1,2^, the number of patients with osteoporosis is increased in postmenopausal women, whose bone resorption activity is promoted along with the reduction of oestrogen. Approximately 50% of 65-year-old women have some experience of fractures due to postmenopausal osteoporosis at some point in their life^3^. Although hormone replacement therapy with oestrogen is effective for postmenopausal osteoporosis, there are risks of uterine and breast cancers. Selective oestrogen receptor modulators have been applied clinically instead of oestrogen, but it is necessary to continue taking these for a long time. Therefore, a novel therapeutic method that balances bone formation and resorption is urgently required. Regenerative medicine using bone marrow-derived mesenchymal stem cells (BM-MSCs), which may possess the ability to control both bone formation and resorption, has thus been focused on as an attractive method to meet these requirements.

MSCs are considered a highly useful cell source for regenerative medicine because of their multi-potentiality and safety profile. In particular, MSCs have been focused upon because of their strong capacity for self-renewal, pluripotency, reduced antigenicity, immunoregulatory functions, and ease of isolation and *in vitro* culture to obtain large numbers of cells for treatment^4^. Significant efforts have already been made for their clinical application as a result of their safety and efficacy in systemic administration^5^. Several clinical trials investigating BM-MSC cell therapies have been reported for autoimmune diseases^6,7^, chronic inflammatory disease^8,9^, myocardial infarction^10^, spinal cord injury^11^, and osteoporosis^12^.

Autologous transplantation of BM-MSCs has great benefits because of a low risk of rejection and exogenous infection, as well as the availability of a stable source of MSCs. However, several functional abnormalities of BM-MSCs have been reported in osteoporosis patients^13–15^, which suggested that BM-MSCs derived from patients are unsuitable for cell therapy. Zhao and others reported that oestrogen potentially regulates the osteoblastic differentiation of human BM-MSCs via PI3K signalling or upregulation of oestrogen receptor alpha, which results in the diminished production of osteoblasts and excessive differentiation of adipocytes from BM-MSCs in postmenopausal osteoporosis patients. Li and others reported that BM-MSCs derived from osteoporosis rats had decreased proliferation ability and pluripotency-related gene expression compared with BM-MSCs derived from normal rats^16,17^. However, the abnormalities of BM-MSCs with respect to regulation of osteoclast activity have rarely investigated. Furthermore, the therapeutic effect of abnormal BM-MSCs on osteoporosis *in vivo* has yet to be clarified. Therefore, we first aimed to investigate whether abnormal BM-MSCs derived from an oestrogen-deficient osteoporosis model exhibit sufficient therapeutic effects on osteoporosis *in vivo*, and then clarified the abnormalities of BM-MSCs by focusing on the regulation of osteoclast activity *in vitro*.

To carry out autologous transplantation using abnormal BM-MSCs, these cells need to be remade into functional cells capable of exerting therapeutic effects on abnormal bone metabolism. In this study, we focused on a novel activator, human UC extract, which we named “Wharton’s jelly extract supernatant” (WJS). The UC is composed of embryonic tissues including umbilical vessels, Wharton’s jelly (WJ), and amniotic membranes. We have elucidated the effect of WJS on the functional improvement of impaired BM-MSCs with diabetes^18^. WJS contained a variety of growth factors, cytokines, extracellular matrix (ECM) proteins and micro-vesicles. WJS improved not only morphological and functional abnormalities of BM-MSCs but also the therapeutic effect on diabetic nephropathy. Moreover, An *et al*. reported that human UC blood-derived MSCs ameliorated bone mineral density in ovariectomised (OVX) nude mice^19^. Therefore, we hypothesised that these biological components might also activate abnormal BM-MSCs derived from an osteoporosis. Thus, we first aimed to investigate the efficacy of WJS on abnormal BM-MSCs *in vitro*. Finally, we investigated the therapeutic effects of activated BM-MSCs on the OVX model *in vivo*.

Our method may allow for autologous cell transplantation using a patient’s own BM-MSCs not only for treatment of osteoporosis, but also other diseases in which autologous BM-MSCs are abnormal.

## Results

### BM-MSCs derived from sham-operated rats (Sham-MSCs) but not OVX rats (OVX-MSCs) ameliorated osteoporosis in OVX rats

**Figure 1:**
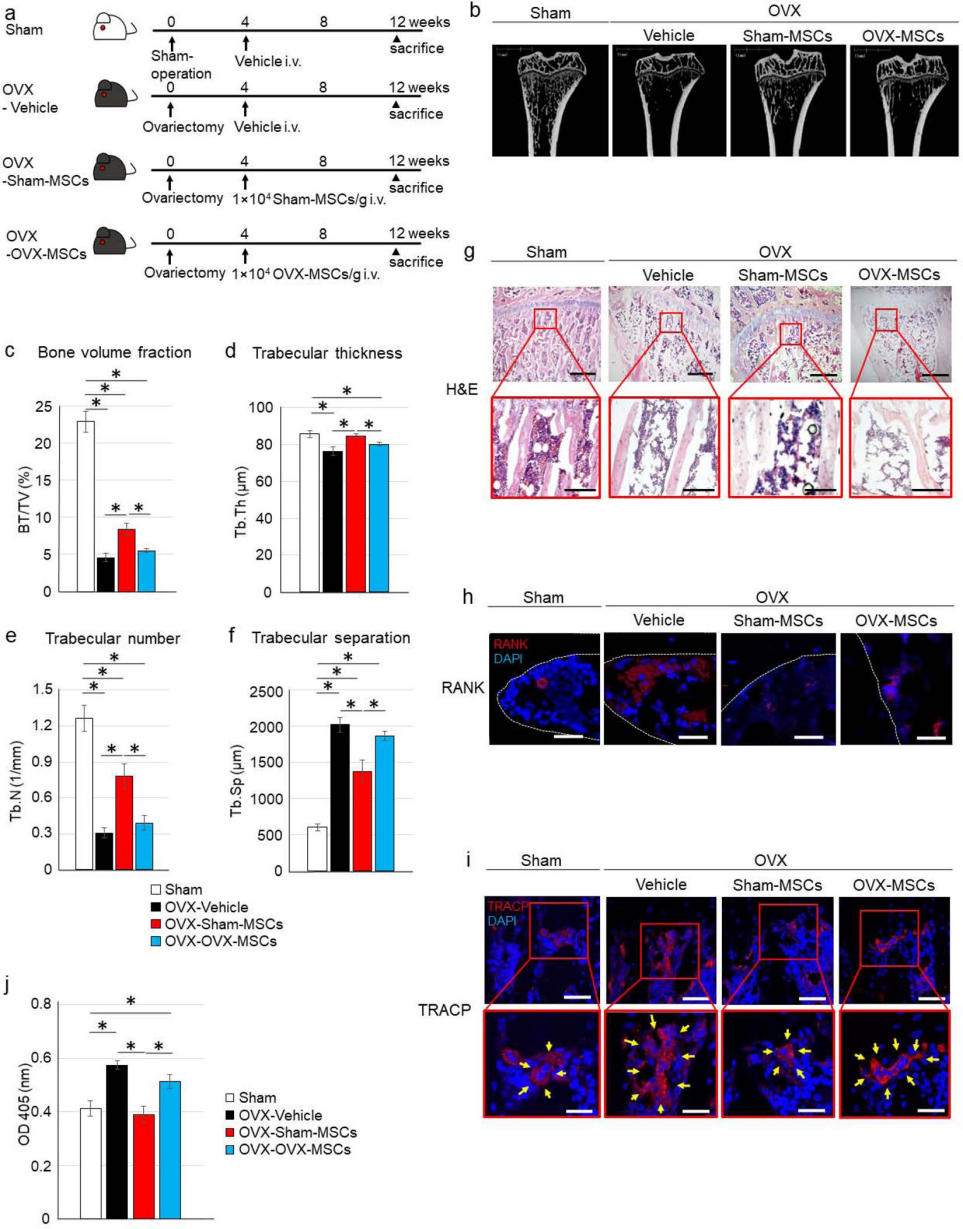
Therapeutic effect of Sham-MSCs and OVX-MSCs in OVX rats. (**a**) Experimental protocol for Sham and OVX rats treated with Vehicle, Sham-MSCs, or OVX-MSCs. (**b**) Representative micro-CT images of tibias. (**c**–**f**) Quantitative changes in trabecular parameters, including trabecular bone volume, expressed as c: percentage of total tissue volume (BV/TV), d: trabecular thickness (Tb.Th), (**e**) Trabecular number (Tb.N), and (**f**) Trabecular separation (Tb.Sp). *P < 0.05. Data are expressed as mean ± SE of 4–5 animals. (**g**) Histological findings of the tibia in H&E-stained sections. Bar: 500 μm in upper panels, 100 μm in lower panels. (**h**) Immunofluorescence staining of the tibia with an anti-RANK antibody (red). DAPI was used for counterstaining nuclei (blue). Bar: 25 µm. (**i**) Immunofluorescence staining of the tibia with an anti-TRACP antibody (red). DAPI was used for counterstaining nuclei (blue). Bar: upper 50 μm, lower 25 µm. (**j**) Serum TRACP levels at the time of sacrifice of rats. *P < 0.05. Data are expressed as mean ± SE of 4–5 animals.

Four weeks after OVX, rats were used as a model of osteoporosis (OVX rats) to perform cell therapy using various types of BM-MSCs. At this time point, a significant decrease of bone volume (Supplementary Fig. S1) indicated successful establishment of the OVX model. The experiment, carried out as shown in Fig. 1a, examined OVX rats treated with Vehicle (OVX-Vehicle), Sham-MSCs (OVX-Sham-MSCs), or OVX-MSCs (OVX-OVX-MSCs). Micro-computed tomography (micro-CT) showed that the number and volume of trabeculae were obviously reduced in the proximal tibia of OVX-Vehicle rats compared with Sham rats (Fig. 1b). In quantitative analysis of micro-CT images, bone volume fraction, trabecular thickness, and trabecular number were significantly decreased, and trabecular separation was significantly increased in OVX-Vehicle rats compared with Sham rats (*P* = 0.002, Fig. 1c; *P* = 0.02, Fig. 1d; *P* = 0.005, Fig. 1e; *P* = 0.005, Fig. 1f). As evaluated by these indicators, Sham-MSCs inhibited the progression of osteoporosis in the proximal tibia of OVX-Sham-MSCs rats compared with OVX-Vehicle rats (*P* = 0.008, Fig. 1c; *P* = 0.03, Fig. 1d; *P* = 0.014, Fig. 1e; *P* = 0.017, Fig. 1f). Conversely, OVX-MSCs did not exhibit adequate therapeutic effects in OVX rats similar to OVX-Vehicle rats, and in contrast to Sham-MSCs (*P* = 0.026, Fig. 1c; *P* = 0.019, Fig. 1d; *P* = 0.025, Fig. 1e; *P* = 0.044; Fig. 1f). Histological findings of haematoxylin and eosin (H&E) staining in the proximal tibia showed similar changes as observed in micro-CT. Specifically, narrowing of trabecular bone and numerous fat deposits in the bone marrow cavity were observed in OVX-Vehicle and OVX-OVX-MSC rats (Fig. 1g). Administration of Sham-MSCs improved these histological changes in OVX rats (Fig. 1g). Notably, the expression of receptor activator of nuclear factor κ-B (RANK) in osteoclasts was increased in OVX-Vehicle rats (Fig. 1h), as was the number of tartrate-resistant acid phosphatase (TRACP)-positive osteoclasts (Fig. 1i). In addition, both of them were decreased in OVX-Sham-MSCs rats compared with OVX-Vehicle rats, while these features were unchanged in OVX-OVX-MSCs rats (Fig. 1h,i). The average size of osteoclasts was larger in OVX-Vehicle rats compared with Sham rats, as determined by the intensity and area of TRACP expression, while it was smaller in OVX-Sham-MSC rats compared with OVX-Vehicle rats. Conversely, OVX-MSCs did not suppress the increased size of osteoclasts (Fig. 1i, right panels). Serum TRACP levels were also significantly higher in OVX-Vehicle rats and OVX-OVX-MSCs rats compared with Sham rats (*P* = 0.011, OVX-Vehicle vs. Sham; *P* = 0.047, OVX-OVX-MSCs vs. Sham; Fig. 1j), while they were significantly lower in OVX-Sham-MSCs rats compared with OVX-Vehicle and OVX-OVX-MSC rats 8 weeks after the administration of each type of BM-MSC (*P* = 0.004, OVX-Sham-MSC vs. OVX-Vehicle; *P* = 0.022, OVX-Sham-MSCs vs. OVX-OVX-MSCs; Fig. 1j).

### WJS improved cell morphology and proliferation abnormalities of OVX-MSCs

**Figure 2:**
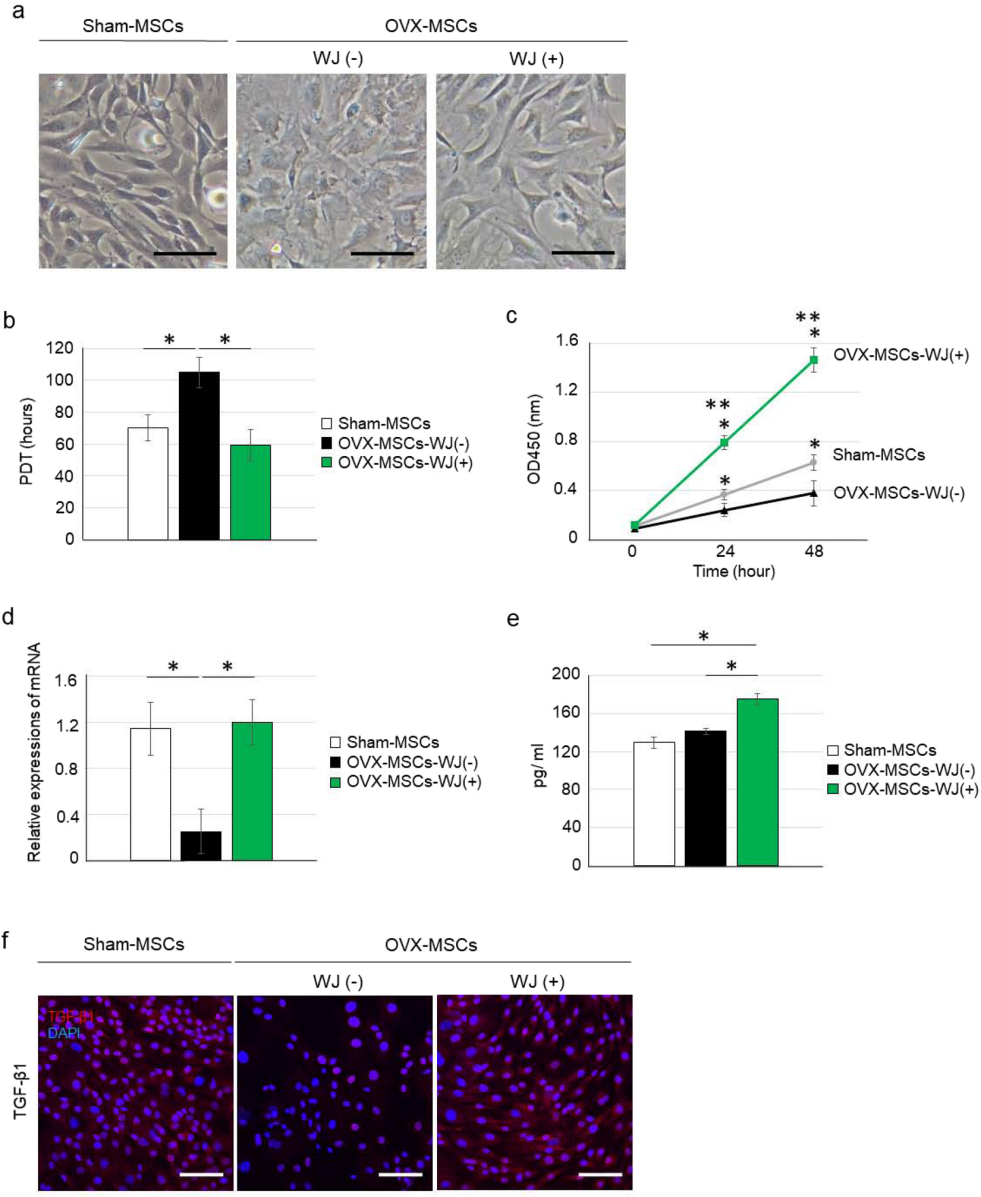
Abnormalities and activating effects of WJS for cell morphology, proliferation, secretion, and protein expression of OVX-MSCs. (**a**) Phase contrast observations of Sham-MSCs (left panel), OVX-MSCs-WJ(-) (middle panel) and OVX-MSCs-WJ(+) (right panel). Images were obtained at 48 h after activation with WJS. Bar: 100 μm. (**b**) Population doubling time of Sham-MSCs, OVX-MSCs-WJ(-), and OVX-MSCs-WJ(+) at passage 2. *P < 0.05. Data are expressed as mean ± SE of 5–6 BM-MSC cultures. (**c**) MTT proliferation assay of Sham-MSCs, OVX-MSCs-WJ(-), and OVX-MSCs-WJ(+). *P < 0.05. Data are expressed as mean ± SE of 5–6 BM-MSC cultures. (**d**) Relative mRNA expression of Sham-MSCs, OVX-MSCs-WJ(-), and OVX-MSCs-WJ(+). *P < 0.05. Data are expressed as mean ± SE of 4 BM-MSCs. *Opg*, osteoprotegerin. (**e**) OPG levels in the culture supernatant of Sham-MSCs, OVX-MSCs-WJ(-), and OVX-MSCs-WJ(+). *P < 0.05. Data are expressed as mean ± SE of 4 BM-MSC cultures. (**f**) Immunofluorescence staining of Sham-MSCs (left panel), OVX-MSCs-WJ(-) (middle panel), and OVX-MSCs-WJ(+) (right panel) with an anti-TFG-β antibody (red). DAPI was used for counterstaining nuclei (blue). Bar: 100 μm.

Morphological findings of OVX-MSCs were abnormal in phase contrast observations, as cells exhibited short and dull cell protrusions, enlarged areas, flat shape, and disordered orientation compared with Sham-MSCs (Fig. 2a). Similar findings were observed from passage 0 (P0) to P2 (Supplementary Fig. S2b). Proliferation of OVX-MSCs was significantly reduced compared with Sham-MSCs, as population doubling time (PDT) at P2 in OVX-MSCs was significantly increased compared with Sham-MSCs (*P* = 0.012, Fig. 2b). Cell growth of OVX-MSCs, as indicated by an MTT proliferation assay, was significantly decreased compared with Sham-MSCs (*P* = 0.041 at 24 h, *P* = 0.036 at 48 h, Fig. 2c).

Next, OVX-MSCs were cultured with an appropriate concentration of WJS for 48 h. In phase contrast observations, OVX-MSCs cultured with WJS [OVX-MSCs-WJ(+)] exhibited morphological changes including thinner and longer cell protrusions, reduced cell area, and spindle shapes similar to Sham-MSCs (Fig. 2a). The results of PDT and MTT proliferation assays indicated the proliferative ability of OVX-MSCs-WJ(+) was significantly improved compared with OVX-MSCs cultured without WJS [OVX-MSCs-WJ(−)] (*P* = 0.013, Fig. 2b; *P* < 0.001 at 24 h, *P* < 0.001 at 48 h, Fig. 2c).

### WJS improved osteoprotegerin (OPG) secretion and transforming growth factor β1 (TGF-β1) expression in OVX-MSCs

Relative osteoprotegerin (*Opg*) mRNA expression in OVX-MSCs was downregulated compared with Sham-MSCs (*P* = 0.023, Fig. 2d). Addition of WJS improved relative *Opg* mRNA expression [*P* = 0.013, OVX-MSCs-WJ(+) vs. OVX-MSCs-WJ(−), Fig. 2d] and OPG secretion [*P* < 0.001, OVX-MSCs-WJ(+) vs. OVX-MSCs-WJ(−); *P* < 0.001, OVX-MSCs-WJ(+) vs. Sham-MSCs; Fig. 2e], as indicated by the concentration of OPG in the supernatant of each BM-MSC type. Expression of transforming growth factor β1 (TGF-β1) in the cytoplasm was downregulated in OVX-MSCs-WJ(−) compared with Sham-MSCs (Fig. 2f). Addition of WJS improved TGF-β1 expression in OVX-MSCs-WJ(+).

### WJS improved OVX-MSC cell mobilisation *in vitro*

**Figure 3:**
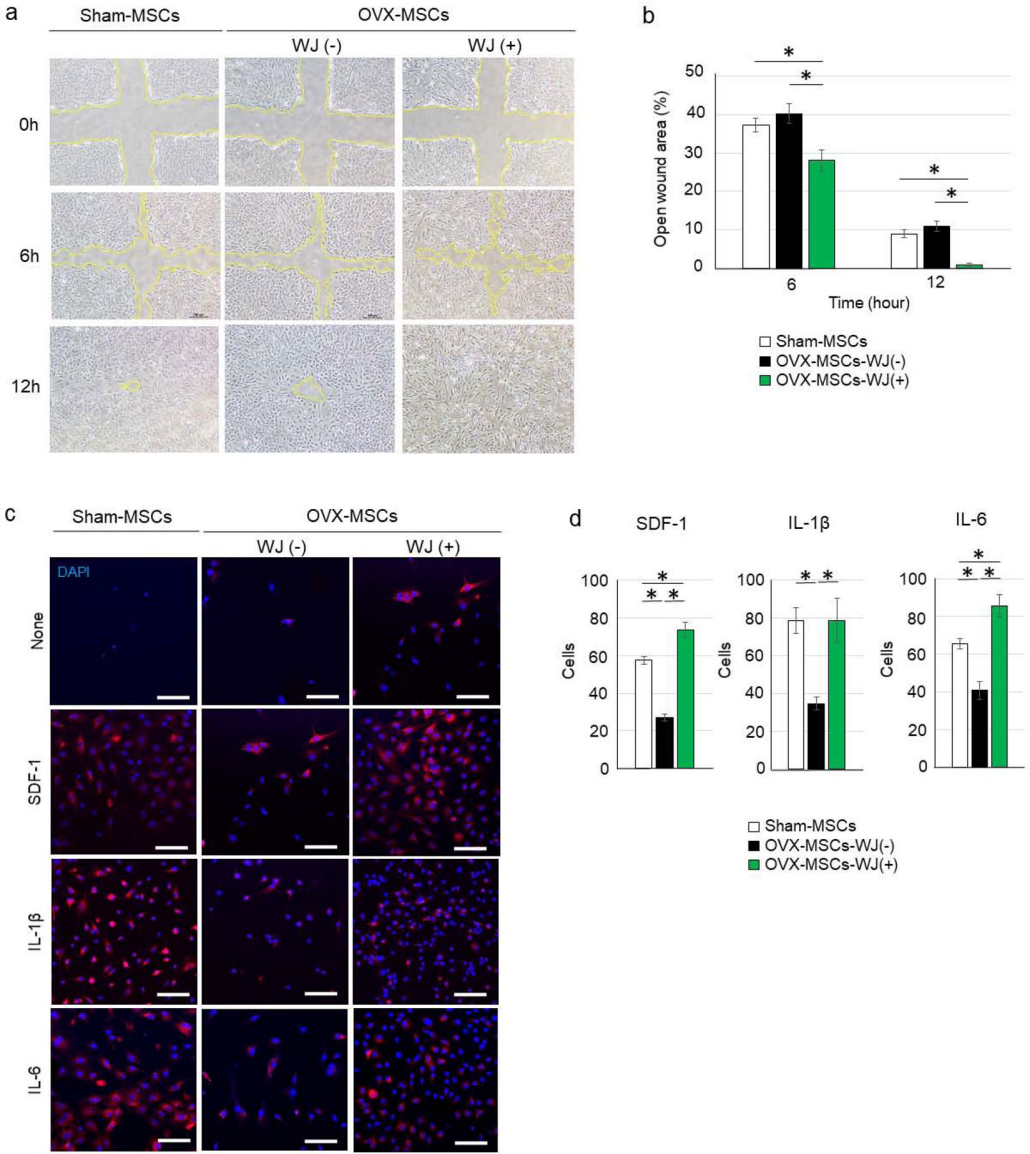
Activating effects of WJS on migration capacity of OVX-MSCs. (**a**) Wound assay of Sham-MSCs, OVX-MSCs-WJ(-), and OVX-MSCs-WJ(+). *P < 0.05. Data are expressed as mean ± SE of 4 BM-MSC cultures. The open wound area was measured at 6 h and 12 h after performing the cross-scratch. Yellow lines indicate the boundary of cell migration. (**b**) The ratio of open wound area at 6 h and 12 h compared with 0 h. Six cross-scratch points in each BM-MSC culture were measured. *P < 0.05. Data are expressed as mean ± SE of 4 BM-MSC cultures. (**c**) Chemotaxis assay of Sham-MSCs, OVX-MSCs-WJ(-), and OVX-MSCs-WJ(+). BM-MSCs induced to migrate to the opposite side of the membrane by SDF-1, IL-1β, and IL-6 as observed by confocal microscope. DAPI was used for counterstaining nuclei (blue). Bar: 100 μm. SDF-1, stromal cell-derived factor 1; IL-1β, interleukin-1 beta; IL-6, interleukin-6. (**d**) Numbers of BM-MSCs migrated to the opposite side of the membrane were counted in three fields of view for each BM-MSC type. *P < 0.05. Data are expressed as mean ± SE of 3 BM-MSC cultures.

Cell mobilisation was reduced in OVX-MSCs-WJ(−) compared with Sham-MSCs, and markedly upregulated in OVX-MSC-WJ(+) as assessed by scratch assay (Fig. 3a). The open wound area was significantly decreased in OVX-MSC-WJ(+) compared with OVX-MSC-WJ(−) 6 h and 12 h after creating the wound (*P* = 0.017 at 6 h, *P* < 0.001 at 12 h, Fig. 3b). Moreover, the open wound area was significantly decreased in OVX-MSC-WJ(+) compared with Sham-MSCs 6 h and 12 h after wound creation (*P* = 0.049 at 6 h, *P* < 0.001 at 12 h, Fig. 2e).

### Stromal-derived factor-1 (SDF-1), interleukin-1 beta (IL-1β), and interleukin-6 (IL-6) induced BM-MSC mobilisation *in vitro*

SDF-1 at 100 ng/ml, IL-1β at 100 ng/ml, and IL-6 at 100 ng/ml induced PKH-26 fluorescence-labelled BM-MSC mobilisation to the opposite side of the transwell membrane (Fig. 3c) and the bottom of culture wells (Supplementary Fig. S3a,b). The number of cells mobilised was smaller in OVX-MSC-WJ(−) compared with Sham-MSCs for each factor examined (SDF-1, IL-1β and IL-6; *P* < 0.001 each, Fig. 3d). In contrast, the number of migrated cells was significantly increased in OVX-MSC-WJ(+) compared with OVX-MSC-WJ(−) for each factor (SDF-1 and IL-6, *P* < 0.001; IL-1β, *P* = 0.002; Fig. 3d). Interestingly, the number of migrated cells was significantly increased in OVX-MSC-WJ(+) compared with Sham-MSCs for SDF-1 and IL-6 (SDF-1, *P* = 0.002; IL-6, *P* < 0.001; Fig. 3d). Mobilised cells were atrophied by exposure to IL-1β and IL-6.

### OVX-MSCs-WJ(+) ameliorated osteoporosis in OVX rats

**Figure 4:**
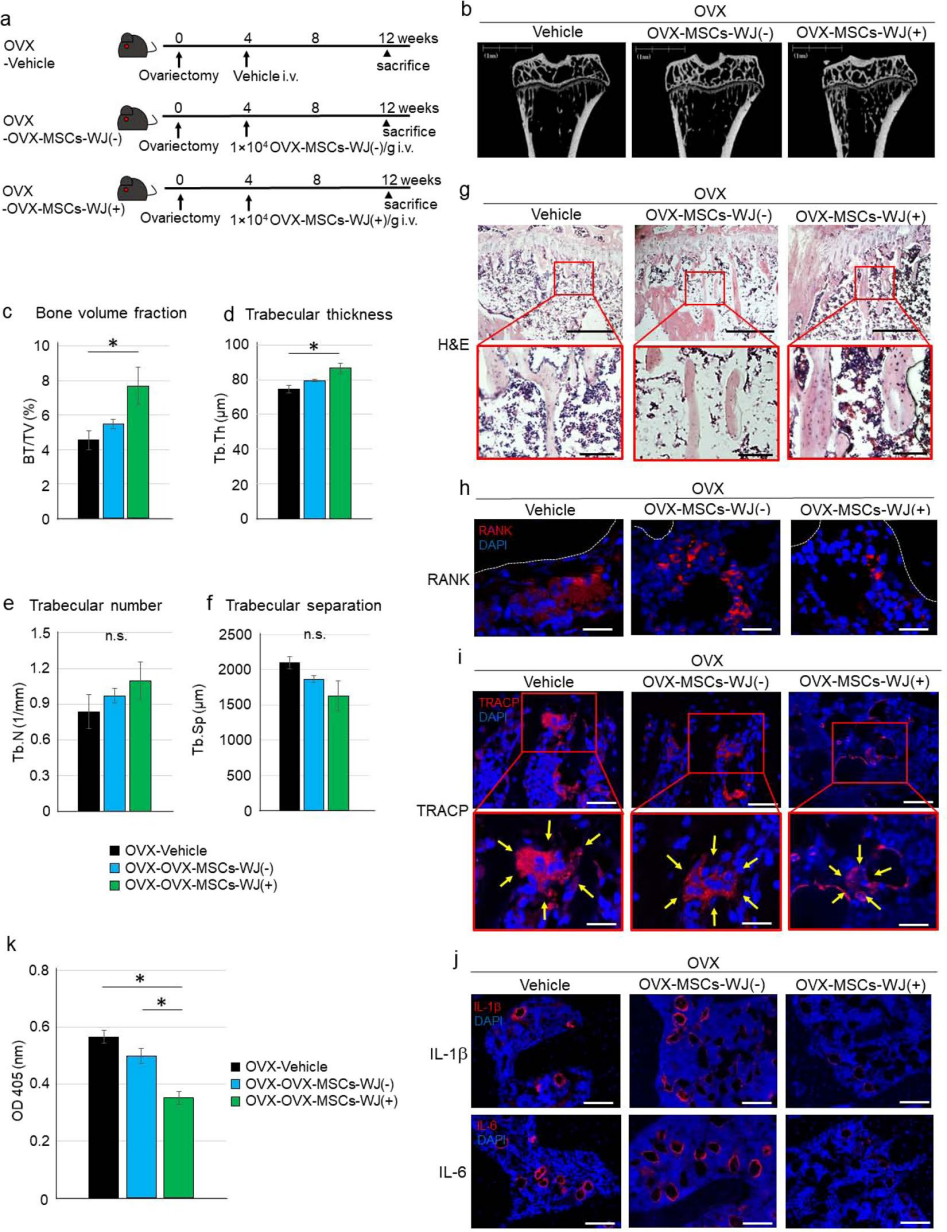
Therapeutic effect of OVX-MSCs activated with WJS in OVX rats. (**a**) Experimental protocol for Vehicle, OVX-MSC-WJ(-), and OVX-MSC-WJ(+) therapies in OVX rats. (**b**) Representative micro-CT images of tibias. (**c**–**f**) Quantitative changes in trabecular parameters, including trabecular bone volume, expressed as c: percentage of total tissue volume (BV/TV), d: trabecular thickness (Tb.Th), (**e**) Trabecular number (Tb.N), and (**f**) Trabecular separation (Tb.Sp). *P < 0.05. Data are expressed as mean ± SE of 4–5 animals. (**g**) Histological findings of the tibia in H&E-stained sections at 8 weeks after administration of Vehicle, OVX-MSC-WJ(-), or OVX-MSC-WJ(+) therapies in OVX rats. Bar: 500 μm in upper panel, 100 μm in lower panel. (**h**) Immunofluorescence staining of the tibia with an anti-RANK antibody (red). DAPI was used for counterstaining nuclei (blue). Bar: 25 µm. (**i**) Immunofluorescence staining of the tibia with an anti-TRACP antibody (red). DAPI was used for counterstaining nuclei (blue). Bar: upper 50 μm, lower 25 µm. (**j**) Immunofluorescence staining of the tibia with an anti-IL-1β antibody (red) (upper) and with an anti-IL-6 antibody (red) (lower). DAPI was used for counterstaining nuclei (blue). Bar: 100 μm. (**k**) Serum TRACP levels at 8 weeks after administration of Vehicle, OVX-MSC-WJ(-), and OVX-MSC-WJ(+) therapies in OVX rats. *P < 0.05. Data are expressed as mean ± SE of 4–5 animals.

The experiment carried out as shown in Fig. 4a evaluated bone tissues of rats 8 weeks after the administration of each type of BM-MSC. Micro-CT analysis of the proximal tibia showed that OVX-MSCs-WJ(+) inhibited the progression of osteoporosis (Fig. 4b), as indicated by the high value of trabecular bone volume and thickness in OVX rats compared with OVX-Vehicle rats (*P* = 0.049, Fig. 4c; *P* = 0.027, Fig. 4d). Conversely, OVX-MSCs-WJ(−) did not show an adequate therapeutic effect in OVX rats (Fig. 4b); that is, no significant changes of relevant indicators was observed in OVX rats compared with OVX-Vehicle rats (Fig. 4c,d). Trabecular number and separation were not significantly changed by the administration of OVX-MSCs-WJ(−) or OVX-MSCs-WJ(+) compared with OVX-Vehicle rats.

Histological findings of the tibia in OVX rats showed similar changes as observed in micro-CT. Thinning and narrowing of the trabecular bone and the presence of fat deposits in the bone marrow cavity were observed in OVX-Vehicle rats with H&E staining (Fig. 4g, left panels). While the administration of OVX-MSCs-WJ(+) improved these histological changes (Fig. 4g, right panels), OVX-MSCs-WJ(−) did not improve histologically observed damage (Fig. 4g, middle panels). Expression of RANK was decreased in OVX-OVX-MSCs-WJ(+) rats compared with OVX-Vehicle rats, while it was unchanged in OVX-OVX-MSCs-WJ(−) rats (Fig. 4h). Number of TRACP-positive osteoclasts, expression of TRACP, and average size of osteoclasts were decreased in OVX-OVX-MSCs-WJ(+) rats compared with OVX-Vehicle rats. Conversely, administration of OVX-MSCs-WJ(−) did not suppress the size of osteoclasts or TRACP expression compared with OVX-MSCs-WJ(+) (Fig. 4i). Expression of IL-1β and IL-6 was decreased in OVX-OVX-MSCs-WJ(+) rats compared with OVX-Vehicle rats, while their expression was unchanged in OVX-OVX-MSCs-WJ(−) rats (Fig. 4j). Serum TRACP levels were significantly lower in OVX-OVX-MSCs-WJ(+) rats compared with OVX-Vehicle and OVX-OVX-MSCs-WJ(−) rats 8 weeks after the administration of each type of BM-MSC (*P* = 0.008, OVX-OVX-MSCs-WJ(+) vs. OVX-Vehicle; *P* = 0.033, OVX-OVX-MSCs-WJ(+) vs. OVX-OVX-MSCs-WJ(−); Fig. 4k).

### Distribution of Sham-MSCs, OVX-MSCs-WJ(−) and OVX-MSCs-WJ(+) in OVX rats

PKH26-labelled BM-MSCs were distributed in the bone marrow of the epiphysis in OVX rats (Supplementary Fig. S4a). The number of cells distributed in bone was larger in OVX-Sham-MSCs rats and OVX-OVX-MSCs-WJ(+) rats compared with OVX-OVX-MSCs-WJ(−) rats on day 1, 3, and 7 after each cell administration (Supplementary Fig. S4b). Numerous PKH26-labelled BM-MSCs were distributed in the lung on days 1, 3 and 7 after each cell administration, but there was no difference between BM-MSC types (Supplementary Fig. S4c,d). Only a few cells were distributed in liver and spleen on day 1 and 3 after cell administration (data not shown).

### Sham-MSCs and OVX-MSCs-WJ(+) ameliorated the maturation and excessive activation of macrophage-derived osteoclasts

**Figure 5:**
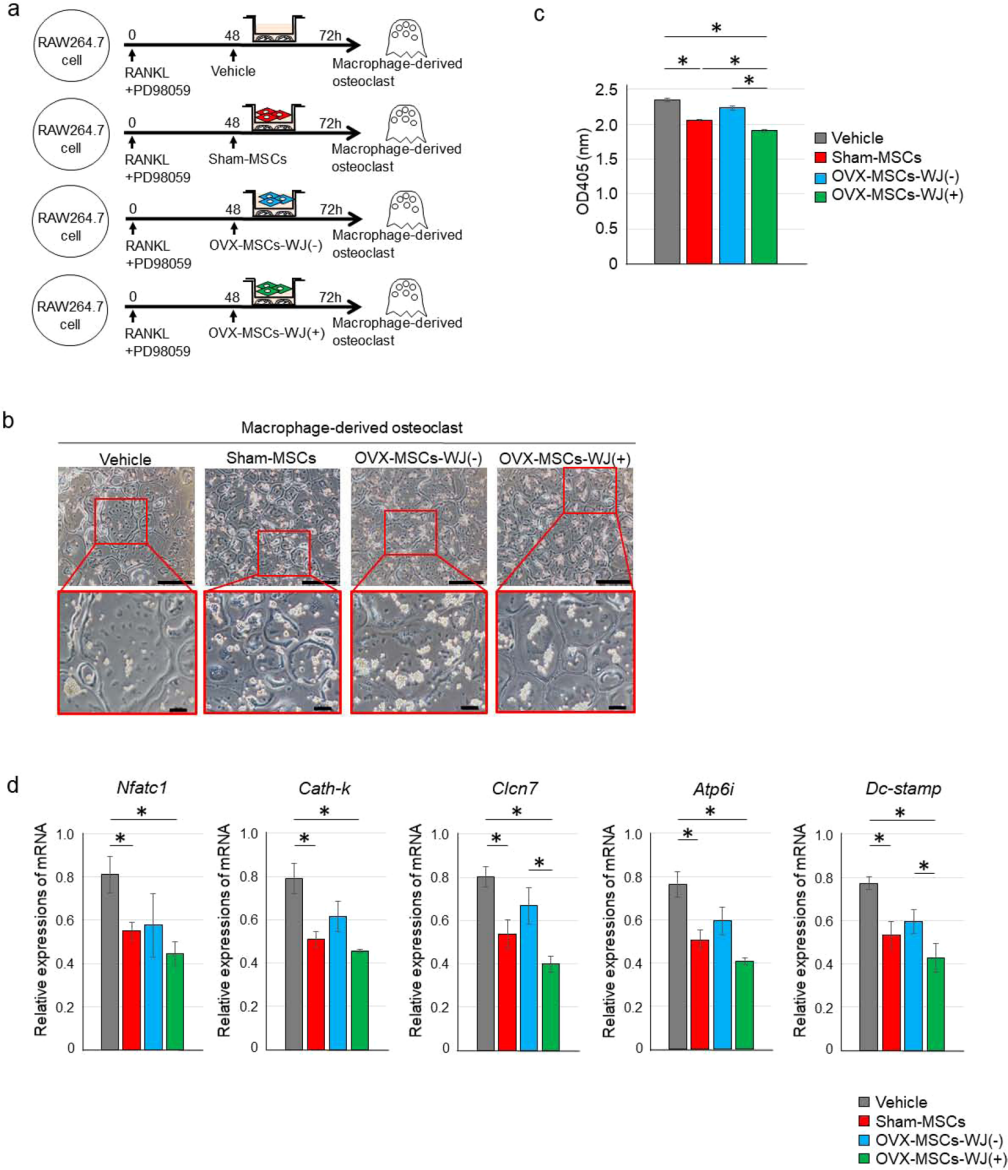
Regulatory effect of OVX-MSCs activated with WJS on RAW264.7 cell-derived osteoclasts. (**a**) Experimental protocol for co-culture of RAW264.7 cell-derived osteoclasts with Vehicle, Sham-MSCs, OVX-MSCs-WJ(-), or OVX-MSCs-WJ(+). (**b**) Phase contrast observations of matured osteoclasts co-cultured without MSCs (left panel) or with Sham-MSCs (middle left panel), OVX-MSCs-WJ(-) (middle right panel), or OVX-MSCs-WJ(+) (right panel) using a Transwell. Images were obtained 72 h after adding RANKL and PD98059 to the culture medium, and 24 h after starting co-culture. Bar: 500 μm in upper panel, 100 μm in lower panel. (**c**) TRACP levels in the supernatant of RAW264.7 cell-derived osteoclasts co-cultured without MSCs and with Sham-MSCs, OVX-MSCs-WJ(-), or OVX-MSCs-WJ(+). Data are expressed as mean ± SE of osteoclasts from 3 experiments. *P < 0.05. (**d**) Relative mRNA expression in RAW264.7 cell-derived osteoclasts. Values represent mean ± SE of osteoclasts co-cultured without MSCs or with Sham-MSCs, OVX-MSCs-WJ(-), or OVX-MSCs-WJ(+) from 3 experiments. *P < 0.05. *Nfatc1*, nuclear factor of activated T cells; *Cath-k*, cathepsin K; *Clc7*, chloride channel-7; *Atp6i*, ATPase, H^+^ transporting, (vacuolar proton pump) member I; *Dc-stamp*, dendritic cell-specific transmembrane protein.

As the number and activity of osteoclasts in bone tissue were suppressed by Sham-MSCs and OVX-MSCs-WJ(+), we investigated whether regulation of osteoclast activity by BM-MSCs was the mechanism of MSC therapy. The macrophage cell line RAW264.7 was differentiated into macrophage-derived osteoclasts by adding receptor activator of nuclear factor κ-B ligand (RANKL) alone or in combination with the MEK inhibitor PD98059 *in vitro* (Supplementary Fig. S5a). Morphologically, macrophages were fused into multinucleated osteoclasts, which became larger and more mature with the addition of RANKL and PD98059 in combination (Supplementary Fig. S5b). TRACP activity in the culture supernatant of macrophage-derived osteoclasts was increased 72 h after induction with RANKL and PD98059 in combination than RANKL alone (*P* < 0.001, RANKL vs. None; *P* < 0.001, RANKL + PD98059 vs. None; *P* < 0.001, RANKL + PD98059 vs. RANKL; Supplementary Fig. S5c). Next, macrophage-derived osteoclasts and each type of BM-MSC were co-cultured indirectly for 24 h (Fig. 5a). The increased average size of osteoclasts was suppressed by co-culturing with Sham-MSCs or OVX-MSCs-WJ(+) compared with Vehicle and OVX-MSCs-WJ(−) (Fig. 5b). In addition, TRACP activity in the culture supernatant was significantly suppressed by co-culture with Sham-MSCs or OVX-MSCs-WJ(+) compared with Vehicle [*P* = 0.002, Sham-MSCs vs. Vehicle; *P* < 0.001, OVX-MSCs-WJ(+) vs. Vehicle; Fig. 5c]. In addition, TRACP activity was significantly downregulated by OVX-MSCs-WJ(+) compared with Sham-MSCs and OVX-MSCs-WJ(−) [*P* = 0.036, OVX-MSCs-WJ(+) vs. Sham-MSCs; *P* = 0.002, OVX-MSCs-WJ(+) vs. OVX-MSCs-WJ(−); Fig. 5c].

### Sham-MSCs and OVX-MSCs-WJ(+) improved excessive expression of an osteoclast fusion-promoting factor and activating factors in macrophage-derived osteoclasts

Relative mRNA expression of various factors that promote osteoclast activity (i.e. *Nfatc1*, *cathepsin-k*, *Clcn7*, and *Atp6i*) and formation (i.e. *Dc-stamp*) was significantly suppressed by Sham-MSCs (*P* = 0.021, *Nfatc1*; *P* = 0.009, *cathepsin-k*; *P* = 0.007, *Clcn7*; *P* = 0.008, *Atp6i*; *P* = 0.006, *Dc-stamp*; Fig. 5d) and OVX-MSCs-WJ(+) (*P* = 0.014, *Nfatc1*; *P* = 0.016, *cathepsin-k*; *P* < 0.001, *Clcn7*; *P* = 0.009, *Atp6i*; *P* = 0.006, *Dc-stamp*; Fig. 5d) compared with Vehicle. These expression was not suppressed by OVX-MSCs-WJ(−). In addition, the expression of *Clcn7* and *Dc-stamp* was significantly downregulated by OVX-MSCs-WJ(+) compared with OVX-MSCs-WJ(−) (*P* = 0.032, *Clcn7*; *P* = 0.047, *Dc-stamp*; Fig. 5d).

### Sham-MSCs and OVX-MSCs-WJ(+) inhibited maturation and excessive activation of primary mouse bone marrow cell (BMC)-derived osteoclasts

**Figure 6:**
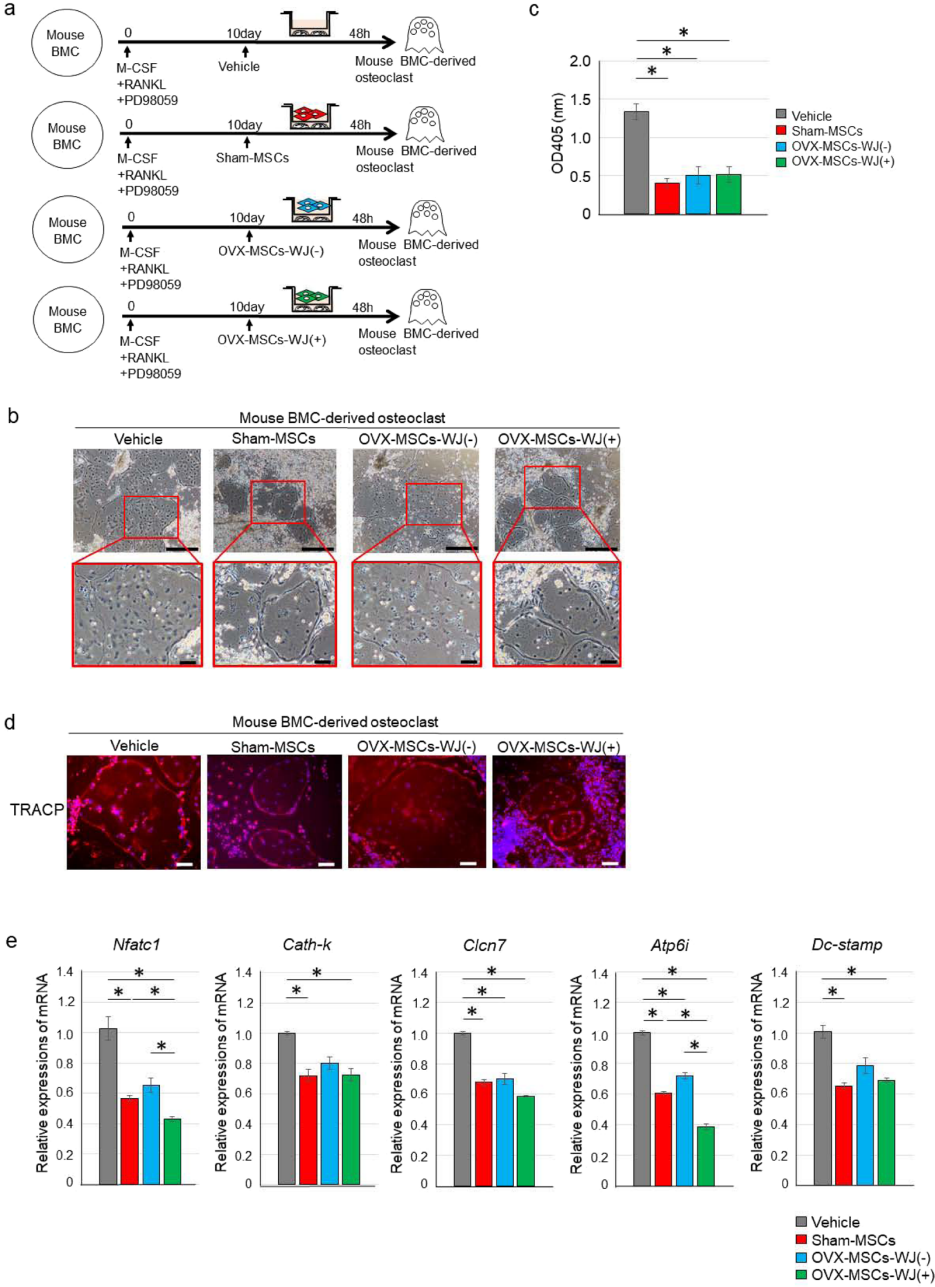
Regulatory effect of OVX-MSCs activated with WJS on mouse bone marrow cell-derived osteoclasts. (**a**) Experimental protocol for co-culture of mouse BMC-derived osteoclasts with Vehicle, Sham-MSCs, OVX-MSCs-WJ(-), or OVX-MSCs-WJ(+). (**b**) Phase contrast observations of matured osteoclasts co-cultured without MSCs (left panel) or with Sham-MSCs (middle left panel), OVX-MSCs-WJ(-) (middle right panel), or OVX-MSCs-WJ(+) (right panel) using a Transwell. Images were obtained 10 days after adding RANKL and PD98059 to the culture medium, and 48 h after starting co-culture. Bar: 500 μm in upper panel, 100 μm in lower panel. (**c**) TRACP levels in the supernatant of mouse BMC-derived osteoclasts co-cultured without MSCs and with Sham-MSCs, OVX-MSCs-WJ(-), or OVX-MSCs-WJ(+). Data are expressed as mean ± SE of osteoclasts from 4–5 experiments. *P < 0.05. (**d**) Immunofluorescence staining of mouse BMC-derived osteoclasts with an anti-TRACP antibody (red). DAPI was used for counterstaining nuclei (blue). Bar: 100 μm. (**e**) Relative mRNA expression in mouse BMC-derived osteoclasts. Values represent mean ± SE of osteoclasts co-cultured without MSCs and with Sham-MSCs, OVX-MSCs-WJ(-), or OVX-MSCs-WJ(+) from 4–5 experiments. *P < 0.05. *Nfatc1*, nuclear factor of activated T cells; *Cath-k*, cathepsin K; *Clc7*, chloride channel-7; *Atp6i*, ATPase, H^+^ transporting, (vacuolar proton pump) member I; *Dc-stamp*, dendritic cell-specific transmembrane protein.

Sham-MSCs and OVX-MSCs-WJ(+) inhibited the maturation and excessive activation of primary osteoclasts. Co-culture experiments performed using primary osteoclasts derived from mouse BMCs cultured with macrophage colony-stimulating factor (M-CSF) showed differentiation into osteoclasts by the addition of RANKL alone or in combination with PD98059 *in vitro* (Supplementary Fig. S6a). Morphologically, osteoclast precursor cells derived from BMCs fused to form multinucleated osteoclasts, which became larger and more mature upon the addition of RANKL and PD98059 in combination (Supplementary Fig. S6b). TRACP activity in the culture supernatant of mouse BMC-derived osteoclasts was increased 10 days after induction with RANKL and PD98059 in combination than RANKL alone (*P* < 0.001, RANKL vs. None; *P* < 0.001, RANKL + PD98059 vs. None; *P* = 0.03, RANKL + PD98059 vs. RANKL; Supplementary Fig. S6c). Next, primary mouse BMC-derived osteoclasts and each type of BM-MSC were co-cultured indirectly for 48 h (Fig. 6a). The increased size of osteoclasts was suppressed by co-culture with Sham-MSCs or OVX-MSCs-WJ(+) compared with Vehicle or OVX-MSCs-WJ(−) (Fig. 6b). TRACP activity in the culture supernatant was significantly suppressed by co-culture with Sham-MSCs, OVX-MSCs-WJ(−), or OVX-MSCs-WJ(+) compared with Vehicle [*P* < 0.001, Sham-MSCs; *P* < 0.001, OVX-MSCs-WJ(−); *P* < 0.001, OVX-MSCs-WJ(+), Fig. 6c]. There was no obvious difference between these BM-MSCs in their ability to inhibit TRACP activity. To clarify the reason why there was no difference in TRACP activity within supernatant between different BM-MSC types, TRACP expression in co-cultured primary mouse BMCs was investigated by immunostaining. TRACP was expressed not only in the margin of matured osteoclasts, but also in small BMCs surrounding the fused osteoclasts (Fig. 6d). That is, TRACP activity within supernatant may not reflect the activity of mature osteoclasts.

### Sham-MSCs and OVX-MSCs-WJ(+) improved excessive expression of an osteoclast fusion-promoting factor and activating factors in primary mouse BMC-derived osteoclasts

Relative mRNA expression levels of osteoclast-related factors in primary osteoclasts was significantly suppressed by Sham-MSCs (*P* = 0.038, *Nfatc1*; *P* = 0.045, *cathepsin-k*; *P* = 0.003, *Clcn7*; *P* < 0.001, *Atp6i*; *P* = 0.001, *Dc-stamp*; Fig. 6e) and OVX-MSCs-WJ(+) (*P* = 0.013, *Nfatc1*; *P* = 0.008, *cathepsin-k*; *P* < 0.001, *Clcn7*; *P* < 0.001, *Atp6i*; *P* = 0.014, *Dc-stamp*; Fig. 6e) compared with Vehicle. Expression of *Clcn7* and *Atp6i* was also downregulated by OVX-MSCs-WJ(−) compared with Vehicle (*P* = 0.031, *Clcn7*; *P* = 0.004, *Atp6i*, Fig. 6e). In addition, the expression of *Nfatc1* and *Atp6i* was significantly downregulated by OVX-MSCs-WJ(+) compared with OVX-MSCs-WJ(−) (*P* = 0.029, *Nfatc1*; *P* < 0.001, *Atp6i*, Fig. 6e).

## Discussion

As BM-MSCs derived from an OVX osteoporosis rat model exhibited reduced therapeutic efficacy, we created a new activation method to improve the therapeutic effects of BM-MSCs for autologous transplantation. We focused on a UC extract, WJS, and demonstrated its ability to improve abnormalities in BM-MSCs arising from oestrogen deficiency. This is the first study to investigate the significant potential of WJS to ameliorate abnormalities in BM-MSCs caused by osteoporosis. BM-MSCs activated by WJS exhibited improved proliferative ability, cell mobilisation, and regulatory effects on the excessive osteolytic properties of osteoclasts.

OVX-MSCs had morphological abnormalities. Cell area was expanded in an irregular manner, with OVX-MSCs exhibiting a flattened appearance and increased cytosolic actin filaments compared with Sham-MSCs, which maintained their slim, spindle shape and uniform cell size. Furthermore, cell proliferation in OVX-MSCs was significantly decreased. The size of BM-MSCs reportedly correlates with their stemness, proliferation, differentiation, and tissue regenerative capacity^20^. Accordingly, abnormalities in the size and shape of OVX-MSCs predicted the following functional disorders of these cells.

Cell mobilisation was downregulated in OVX-MSCs. According to the results of scratch and chemotaxis assays, not only mobilisation itself, but also the response to chemokine stimulants, such as SDF-1, IL-1β and IL-6, were extremely downregulated in OVX-MSCs compared with Sham-MSCs. BM-MSCs are known to migrate in response to many chemotactic factors, including platelet-derived growth factor-AB (PDGF-AB), insulin-like growth factor 1 (IGF-1) and SDF-1^21^. The migratory capacity of BM-MSCs is under the control of receptor tyrosine kinases, growth factors, and CC and CXC chemokines. The mobilisation of MSCs, as well as their subsequent homing to damaged bone tissue, may depend on the systemic and local inflammatory state. Ovariectomy leads to a significant increase in IL-1β and IL-6 levels in tibia^22^ and serum^23^. In our study, expression of IL-1β and IL-6 were increased in the tibias of OVX rats, which might induce enhanced mobilisation of BM-MSCs. However, due to the low reactivity of OVX-MSCs to these stimuli, an insufficient number of cells were able to migrate to local lesions. Reflecting these cell characteristics, the number of OVX-MSCs distributed in the bone epiphysis might contribute to the therapeutic effects of BM-MSCs.

OVX-MSCs could not regulate excessively activated osteoclasts with high osteolytic capacity *in vivo*, as indicated by reduced expression and secretion of OPG. By strongly binding to RANK to form a decoy receptor for RANKL, OPG triggers osteoclast differentiation and subsequently suppresses the activity of the essential transcription factor NFATc1^24^. Expression of TGF-β1, which was also downregulated in OVX-MSCs, is known to increase *Opg* transcription in osteoblasts^25^ and promote osteoblastic cell proliferation, function, and survival^26^, which ultimately encourages bone formation and regulates osteoclast activity. As the expression of OPG and TGF-β1 were downregulated in OVX-MSCs, they could not sufficiently suppress osteoclast activity, which may cause the reduction of bone volume and loss of therapeutic effects observed in OVX rats.

As expected, OVX-MSCs elicited reduced therapeutic effects in OVX rats. Bone strength is decreased in osteoporosis, as indicated by reduced bone volume fraction and bone quality. Bone strength and microstructure have been evaluated by micro-CT in the OVX model^27^, and bone quality can be determined by assessing trabecular thickness, number, and separation. In this study, OVX-MSCs did not improve any of these indicators, while Sham-MSCs improved them sufficiently. This result was consistent with histological findings showing the absence of an improvement of trabecular bone and the presence of fat deposits in the bone marrow cavity. Furthermore, serum TRACP levels and expression of RANK and TRACP in osteoclasts was not sufficiently decreased in OVX rats treated with OVX-MSCs compared with Sham-MSCs *in vivo*. Thus, the reduced therapeutic effects of OVX-MSCs was considered to arise from the functional abnormalities of these cells, as shown *in vitro*.

We developed a UC extract, namely WJS, as an activator of OVX-MSCs to improve their abnormalities. WJS contains various physiologically active substances including growth factors, ECM, amino acids, exosomes, and nucleic acids represented by microRNAs (miRNAs) originating in these tissue components^18,28–30^. In addition, the cellular components of UC, represented by UC-MSCs, produce large amounts of autocrine/paracrine factors, such as IGF-1, basic fibroblast growth factor (b-FGF), TGF-β, PDGF, epidermal growth factor (EGF), and miRNAs^28,31^. Preconditioning BM-MSCs with IGF-1 and b-FGF in combination induces the expression of cell survival-related factors such as IGF-1, FGF-2, Akt, GATA-4, and Nkx 2.5, and downregulates cell senescence- and apoptosis-related factors such as p16^INK4a^, p66, p53, Bax, and Bak^32,33^. PDGF and b-FGF are essential components for the growth-promoting effects of BM-MSCs^34^. EGF induces growth factor production and the paracrine activity of MSCs via EGF-EGF receptor 1 signalling^35^. Recent reports identified critical roles for various miRNAs as regulators of MSCs. Minayi *et al*. reported that miR-210 upregulates the proliferation of BM-MSCs^36^. Lv *et al*. reported that miR-21 suppresses the apoptosis of BM-MSCs via activation of the PI3K/Akt pathway^37^. MiR-148a and miR-148b, which are found in UC blood MSC-derived exosomes, reportedly regulate the proliferation of UC blood MSCs by upregulating NF-κB or hedgehog signalling^38^. Considering these reports, WJS might improve the proliferation and cell senescence of OVX-MSCs, as it contains a cocktail of these cell activation factors.

Morphological and functional abnormalities of OVX-MSCs were improved by WJS. Morphologically, expansion of cell area, flattening, and irregularities in the size and shape of cells were improved. The proliferation of OVX-MSCs was increased by the addition of WJS, as indicated by PDT and MTT assay. An improvement of proliferation is thought to be beneficial in cell therapy to secure an adequate number of cells for transplantation from bone marrow within a short period of time. Cell mobilisation was also improved by the addition of WJS, as indicated by scratch and chemotaxis assays. The improvement of cell mobilisation to damaged tissues would greatly benefit the efficiency of cell therapies by promoting cell distribution. In addition, WJS improved the expression of OPG and TGF-β1 in OVX-MSCs, suggesting OVX-MSCs-WJ(+) more effectively regulate osteoclast activity.

*In vivo* treatment with WJS-activated OVX-MSCs significantly improved trabecular bone volume and thickness as observed with micro-CT. These improvements were consistent with the recovery of histological findings for bone tissues and observed reductions of RANK, TRACP, IL-1β, and IL-6 expression. RANK is a receptor expressed on osteoclast precursor cells that transmits intracellular signals essential for the differentiation and activation of osteoclasts by binding with RANKL. In postmenopausal osteoporosis, bone resorption is also increased by the production of monocyte-related cytokines, such as IL-1, IL-6, and TNF-α, which induce RANKL expression in bone tissue and enhance RANKL-RANK-mediated osteoclastogenesis^39^. Conversely, UC-MSCs are known to exert immunosuppressive effects on monocytes by regulating cytokine production^40^. Considering the similar characteristics of OVX-MSCs-WJ(+) and UC-MSCs, WJS might change the function of OVX-MSCs to assist them in exerting immunoregulatory effects on osteoclasts by regulating RANK signalling with increasing OPG and cytokine production. These findings correlated with decreased TRACP-positive osteoclasts and expression of IL-1β and IL-6 in the OVX-MSCs-WJ(+) group compared with Vehicle and OVX-MSCs-WJ(−) groups *in vivo*.

The therapeutic effects continued for 8 weeks despite the disappearance of the administered cells in bone tissue within few days. Several studies have reported that systemic administration of MSCs effectively prevented bone loss despite poor BMC homing and short-term engraftment MSCs^41,42^. In addition, systemic injection of human umbilical cord blood derived-MSCs and their conditioned medium were effective on OVX-induced bone loss in mice^19^. Based on the previous reports, we speculated two reasons for these phenomena. First, transplanted BM-MSCs migrated to the epiphysis of bone tissues and triggered osteoclast regulation. Once the trigger for repair was turned on, the effect continued even after the cells disappeared. Second, administered BM-MSCs were also distributed to the lung and were still detectable 7 days after transplantation. As we showed that osteoclast activity was regulated by a paracrine effect of BM-MSCs, the therapeutic effect may have been sustained by paracrine factors derived from BM-MSCs remaining in the lung. Moreover, as the levels of OPG secretion and TGF-β expression were higher in Sham-MSCs and OVX-MSCs-WJ(+) compared to OVX-MSCs-WJ(−), the sufficient therapeutic effect was obtained in OVX rats which were treated with these functional BM-MSCs.

OVX-MSCs-WJ(+) suppressed the fusion and enlargement of osteoclasts derived from both RAW cells and primary mouse BMCs. Expression of osteoclast-activating factors (i.e. *Nfatc1*, *cathepsin-k*, *Clcn7*, and *Atp6i*), a fusion-promoting factor (i.e. *Dc-stamp*) and TRACP levels in the culture supernatants of osteoclasts were also suppressed by culture with OVX-MSCs-WJ(+). Significant decreases in *Nfatc1*, a master transcription factor of osteoclasts located downstream of the RANK pathway, *cathepsin-k*, a degradation factor of type 1 collagen, *Clcn7*, a chlorine transporter that promotes bone resorption, *Atp6i*, an acid transporter that promotes bone resorption, and *Dc-stamp*, which specifically promotes the fusion of macrophages and multinucleation of osteoclasts, were observed in osteoclasts cultured with OVX-MSCs-WJ(+) compared with Vehicle. In addition, *Nfatc1* and *Atp6i* were significantly downregulated in mouse BMC-derived osteoclasts, while *Clcn7* and *Dc-stamp* were significantly downregulated in macrophage-derived osteoclasts in response to administration of OVX-MSCs-WJ(+) compared with OVX-MSCs-WJ(−). These differences were thought to arise from the presence of BMCs other than osteoclasts producing physiologically active substances such as cytokines. Considering these findings, OVX-MSCs-WJ(+) successfully regulated osteoclast activity and maturation via different or overlapping mechanisms. As this was observed in the indirect co-culture of BM-MSCs and osteoclasts, a paracrine effect of BM-MSCs may contribute to this observation.

**Figure 7:**
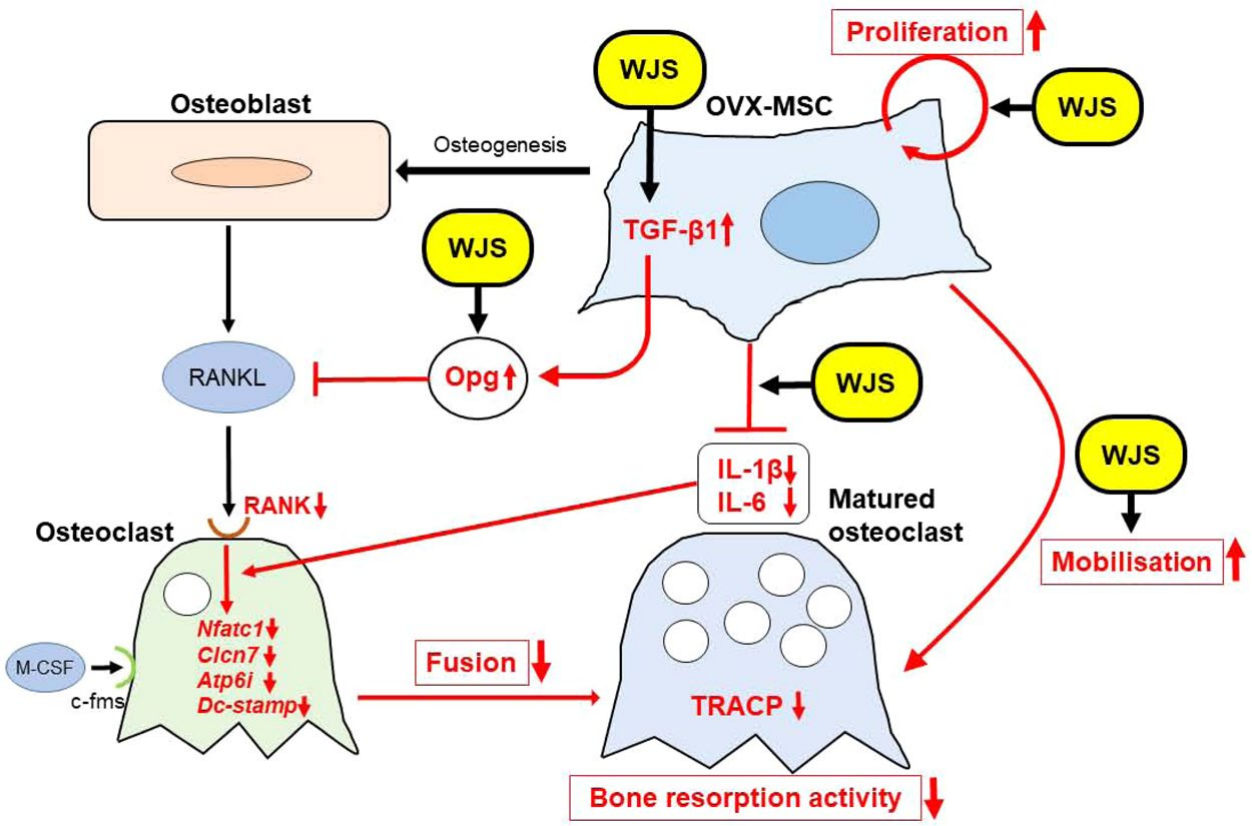
Presumed activation mechanisms of WJS on OVX-MSCs and resulting therapeutic effects for osteoporosis. The site of action of WJS on OVX-MSCs and the resulting therapeutic mechanism for osteoporosis.

In conclusion, we developed a novel method to activate abnormal BM-MSCs derived from a postmenopausal osteoporosis rat model using WJS, and demonstrated the morphological and functional improvement of OVX-MSCs *in vitro*. WJS enhanced the therapeutic effects of OVX-MSCs on OVX rats *in vivo*. The site of action of WJS on OVX-MSCs and their therapeutic mechanism for osteoporosis are shown in Fig. 7. This method may provide great benefit for the autologous transplantation of BM-MSCs in osteoporosis patients not only in the postmenopausal period, but also for bone diseases caused by other pathological mechanisms throughout life.

## Methods

### Animal model of osteoporosis

Eight-week-old female Wistar rats weighing 135–145 g were purchased from Japan SLC, Inc. (Shizuoka, Japan). Rats were housed in a temperature-controlled room (24 ± 1 °C) with a 12-h light/dark cycle and given free access to food and water. Rats received either a sham operation (Sham) or OVX under general anaesthesia. The sham operation was performed using the same surgical procedure as for OVX, but without removing the ovaries. Both Sham and OVX rats underwent minimal surgery through a dorsal approach^43^. All methods for animal experiments were performed in accordance with the relevant guidelines and regulations of the Animal Experiment Committee of Sapporo Medical University (Sapporo, Japan). All experimental protocols and studies were approved by the Animal Experiment Committee of Sapporo Medical University.

### Study design

First, rats were divided into four groups: (1) rats with sham operation (Sham rats; n = 5), (2) OVX rats administered Vehicle (OVX-Vehicle; n = 5), (2) OVX rats administered Sham-MSCs (OVX-Sham-MSCs; n = 5), and (3) OVX rats administered OVX-MSCs (OVX-OVX-MSCs; n = 5). Second, 15 rats were divided into three groups 4 weeks after OVX: (1) OVX rats administered Vehicle (OVX-Vehicle; n = 5), (2) OVX rats administered OVX-MSCs not activated with WJS [OVX-OVX-MSCs-WJ(−); n = 5], and (3) OVX rats administered OVX-MSCs activated with WJ [OVX-OVX-MSCs-WJ(+); n = 5]. These rats were sacrificed 8 weeks after each administration.

### Isolation, culture, and characterisation of BM-MSCs

BMCs were harvested from OVX rats in 12 weeks after OVX (OVX-MSCs) or sham operation (Sham-MSCs) as previously described^44^. BM-MSCs were characterised by assessing their immunophenotype and differentiation potential as described in Supplementary Methods in detail. Primary and secondary antibodies used for fluorescence-activated cell sorting are listed in Supplementary Tables S1 and S2.

### Intravenous administration of BM-MSCs

Four weeks after ovariectomy, OVX rats were administered Vehicle or 1 × 10^4^ Sham-MSCs (OVX-Sham-MSCs), OVX-MSCs [OVX-OVX-MSCs-WJ(−)], or OVX-MSCs activated with WJS [OVX-OVX-MSCs-WJ(+)] per g of body weight via the tail vein. OVX-MSCs-WJ(+) was washed with PBS, centrifuged to isolate cells, and further resuspended in PBS prior to administration to avoid leaving WJS in BM-MSCs.

### Evaluation of bone mass and microarchitecture by micro-CT

The right tibia of each rat was isolated and fixed in 4% ethanol for micro-CT with a ScanXmate-L090 (Comscantecno, Yokohama, Japan) operated at a lamp voltage of 75 kV and current of 100 μA, using the software X sys FP Version 1.7 and coneCTexpressIV 1.54 (Comscantecno). Samples were scanned at a magnification factor of 5.263 and spatial resolution of 19.001 μm/pixel. Captured images were rendered using the machine software TRI/3D BON (Ratoc System Engineering Co., Ltd., Tokyo, Japan).

### Histological findings of bone tissues

The left tibia from each rat was fixed with 4% paraformaldehyde in phosphate-buffered saline and decalcified with 10% ethylenediaminetetraacetic acid. Bone tissues were cut into thin sections (7 µm) and stained with H&E (Wako, Osaka, Japan). Stained sections were observed with a light microscope (NIS element BR 3.0; Nikon, Tokyo, Japan).

### Immunofluorescence staining of bone tissues

Bone tissues were cut into thin sections (7 µm). Immunofluorescence staining of RANK, TRACP, IL-1β, and IL-6 was performed. Bone samples were incubated with primary and secondary antibodies (Supplementary Tables S3 and S4). Nuclei were stained with DAPI (Dojindo Laboratories, Kumamoto, Japan). Stained sections were observed under a confocal laser-scanning microscope (LSM 510; Carl Zeiss, Oberkochen, Germany).

### Measurement of TRACP levels in serum and culture supernatant of osteoclasts

Blood samples were obtained through cardiac puncture at sacrifice, and serum was separated and stored at −80 °C until use. Culture supernatants of osteoclasts were collected and stored at −80 °C until use. TRACP levels in serum and culture supernatants were measured with a TRACP & ALP Assay Kit (Takara Bio, Inc., Shiga, Japan) according to the manufacturer’s instructions.

### Preparation of WJS from UC tissues

WJS was prepared as previously described^18^. Briefly, whole UC tissues were cut into short pieces, and all of the sectioned sheathes of amnion, UC vessels, and WJ were collected. Tissues were suspended in serum-free medium, and the supernatants of tissue suspensions were obtained as WJS. The protein concentration was determined with a bicinchoninic acid (BCA) Protein Assay Kit (Thermo Fisher Scientific, Waltham, MA, USA).

### Activation of OVX-MSCs with WJS

WJS was added to OVX-MSCs at a concentration 0.25 mg/mL. BM-MSCs were analysed for cell morphology, proliferative potential, mobilisation ability, differentiation potential, mRNA expression and protein expression 48 h after WJS administration.

### Phase contrast microscopic observation of BM-MSCs

Morphological findings of BM-MSCs were observed by phase contrast microscopy (Eclipse TE200; Nikon).

### Proliferation assays of BM-MSCs

PDT was measured at P2 and calculated using the formula: PDT = tplg2/(lgNH − lgNI), where NI is the inoculum cell number, NH is the cell harvest number, and t is the time of the culture (in hours). The mean and standard deviation were calculated for three independent experiments. The PDT assay was performed using P2 because cells other than MSCs, such as macrophages, fibrocytes and haematopoietic stem cells, contaminated passages 0 to 1 in rat BMC cultures.

BM-MSCs at P2 were plated in 96-well cell culture plates (Corning Costar; Sigma-Aldrich, St. Louis, MO, USA) at a density of 2.5 × 10^3^ cells/well. Triplicate wells were used for each sample. Proliferation of BM-MSCs was analysed using a Cell Counting Kit-8 (Dojindo Laboratories). The absorbance at 450 nm (OD450) was measured with a microplate reader (Infinite M1000 Pro; TECAN, Männedorf, Switzerland).

### Quantitative real-time reverse transcription polymerase chain reaction (RT-PCR) of BM-MSCs

Quantitative RT-PCR analysis was performed as described in Supplementary Methods in detail. Specific primers for rat *Opg* was used, which are described in Supplementary Table S5. Rat *Gapdh* primers acted as an internal standard for RNA integrity and quantity.

### Measurement of OPG levels in the supernatant of BM-MSCs

After cell activation with or without WJS for 48 h, the medium was replaced with WJ-free medium and cells were cultured for an additional 24 h. The medium was then collected and stored at −80 °C until use. OPG levels in culture supernatants were measured with a Rat Osteoprotegerin (OPG) ELISA Kit (MyBioSource, Inc., San Diego, USA) according to the manufacturer’s instructions.

### Immunofluorescence staining of BM-MSCs

Immunofluorescence staining of TGF-β1 in in each BM-MSC type [Sham-MSCs, OVX-MSCs-WJ(−), and OVX-MSCs-WJ(+)] was performed as described in Supplementary Methods in detail. Primary and secondary antibodies were listed in Supplementary Tables S3 and S4.

### Scratch assay and Chemotaxis assay

Scratch assay and Chemotaxis assay were carried out as described in Supplementary Methods in detail. The mobilisation ability of BM-MSCs was evaluated by measuring the area of an open wound after scratching the cell monolayer. The chemotaxis assay was carried out using culture membrane inserts using 100 ng/ml of SDF-1 (Peprotech, Inc., Rocky Hill, NJ, USA), 100 ng/ml of IL-1β (BioLegend, Inc. San Diego, USA), or 100 ng/ml of IL-6 (BioLegend). Recombinant chemokines used for chemotaxis assay were listed in Supplementary Table S6.

### Regulation of RAW264.7 cell-derived osteoclast activities by BM-MSCs *in vitro*

Osteoclastogenesis of monocytes/macrophages was conducted by modifying the method of Yonezawa *et al*.^45^. The murine RAW264.7 monocyte/macrophage cell line (ATCC, Manassas, VA, USA) was used as osteoclast precursors by employing culture methods described in the Supplementary Methods in detail. Subsequently, RAW264.7 cells were cultured in the presence of RANKL and PD98059 to investigate the functional effect of BM-MSCs on osteoclasts. After 48 h, osteoclastic cells differentiated from RAW264.7 cells were co-cultured indirectly with BM-MSCs. After 24 h of co-culture, cell morphology, supernatant TRACP levels, and osteoclast-related gene expression were evaluated. mRNA expression of osteoclast-related genes was determined by quantitative real-time RT-PCR using primers listed in Supplementary Table S7.

### Regulation of mouse BMC-derived osteoclast activity by BM-MSCs *in vitro*

Osteoclastogenesis of mouse BMCs was conducted by modifying the method of Yonezawa *et al*.^45^. Briefly, mouse BMCs from 7-week-old C57BL/6 mice were harvested as described in the Supplementary Methods for use as osteoclast precursors. To investigate the functional effect of BM-MSCs on osteoclasts, mouse BMCs were cultured in the presence of M-CSF (100 ng/mL), RANKL (100 ng/mL), and PD98059 (20 mM). After 10 days, osteoclastic cells differentiated from mouse BMCs were co-cultured indirectly with BM-MSCs. After 48 h of co-culture, cell morphology, TRACP staining, supernatant TRACP levels, and osteoclast-related genes were evaluated. mRNA expression of osteoclast-related genes was determined by quantitative real-time RT-PCR using primers listed in Supplementary Table S7.

### Ethics committee approval

The human study was conducted in accordance with the ethical principles of the Declaration of Helsinki and approved by the Ethics Committee of Sapporo Medical University (Registration numbers: 24–142, 25–1227, 262–1031, 262–1046, 262–110). Written informed consent was received from participants prior to their inclusion in the study.

### Statistical analysis

Data from quantitative experiments are expressed as mean ± standard error (SE) values. One-way analysis of variance was employed for multiple comparisons. Two-way repeated measures (mixed between-within subjects) analysis of variance followed by Bonferroni’s test was used for serial assessment. Differences were considered significant at *P* < 0.05 in all two-tailed tests. Statistical analysis was performed using SPSS software (version 16.0; SPSS, Inc., Chicago, IL, USA).

## Electronic supplementary material

Supplementary information
