# Author Correction: Umbilical cord extracts improve osteoporotic abnormalities of bone marrow-derived mesenchymal stem cells and promote their therapeutic effects on ovariectomised rats

**DOI:** 10.1038/s41598-020-78836-8

**Published:** 2020-12-09

**Authors:** Akira Saito, Kanna Nagaishi, Kousuke Iba, Yuka Mizue, Takako Chikenji, Miho Otani, Masako Nakano, Kazusa Oyama, Toshihiko Yamashita, Mineko Fujimiya

**Affiliations:** 1 Department of Orthopaedic Surgery, Sapporo Medical University, Sapporo, Japan; 2 Second Department of Anatomy, Sapporo Medical University, Sapporo, Japan; 3 Department of Diabetic Cellular Therapeutics, Sapporo Medical University, Sapporo, Japan

Correction to: *Scientific Reports*, 10.1038/s41598-018-19516-6, published online 18 January 2018

This Article contains errors.

As a result of a figure assembly error, Figure 3C panel None/WJ(+) is a duplication of panel SDF-1/WJ(−). The correct Figure 3 appears below as Figure 1.

**Figure 1:**
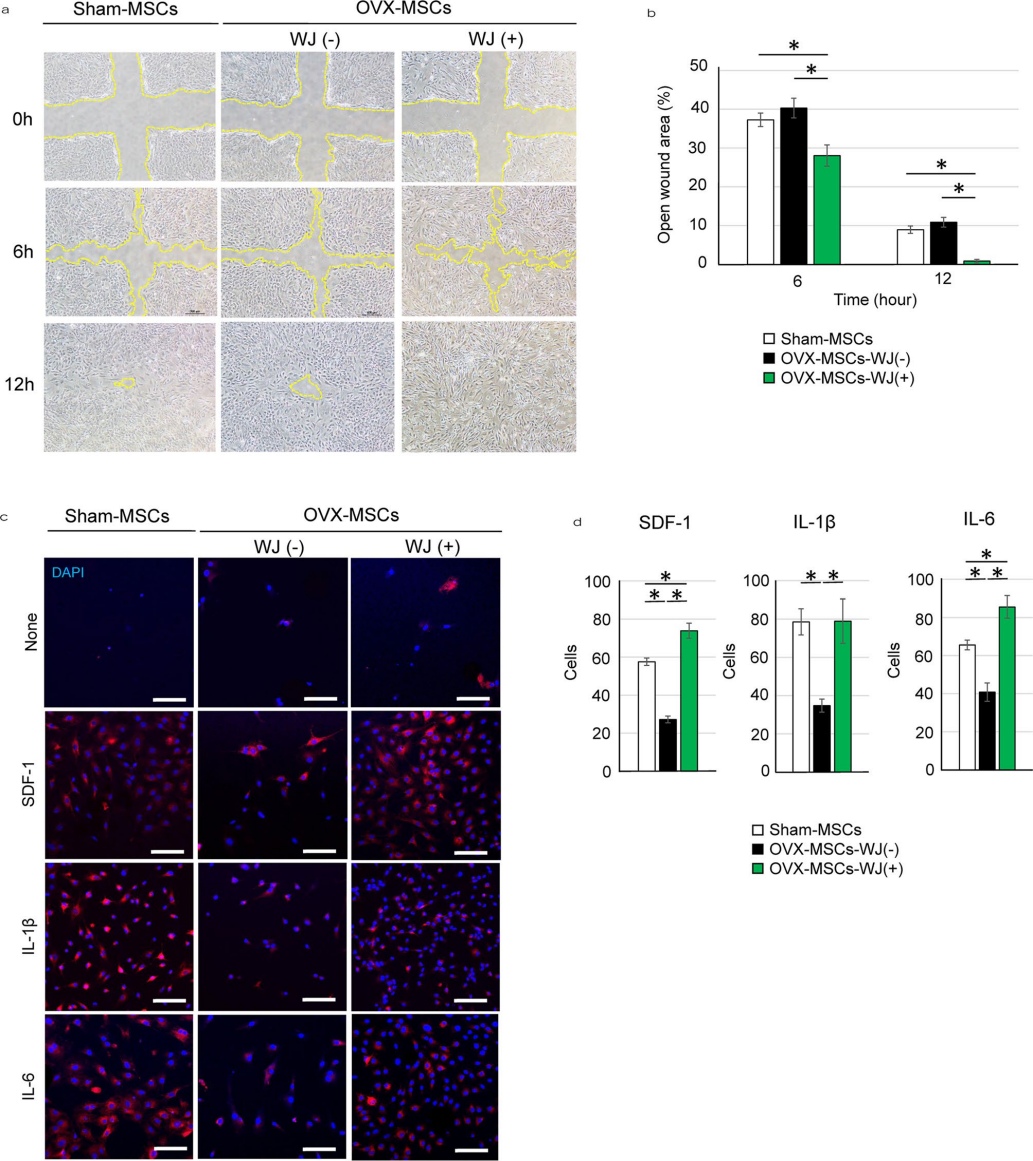
The correct version of Figure 3.

This change does not affect the conclusions of the Article.

