# Supplementary material for: Umbilical cord extracts improve osteoporotic abnormalities of bone marrow-derived mesenchymal stem cells and promote their therapeutic effects on ovariectomised rats
Source: Sci Rep. 2018 Jan 18;8:1161. doi: 10.1038/s41598-018-19516-6 (PMC5773568; doi:10.1038/s41598-018-19516-6)

SUPPLEMENTARY INFORMATION

1. Supplementary Results
2. Supplementary Methods
3. Supplementary Figure and Table Legends
4. Supplementary Tables
5. Supplementary Figures

Title

Umbilical cord extracts improve osteoporosis-induced abnormalities of bone marrow-derived mesenchymal stem cells and promote their therapeutic effects on ovariectomised rats

Akira Saito, Kanna Nagaishi*, Kousuke Iba, Yuka Mizue, Takako Chikenji, Miho Otani, Masako Nakano, Kazusa Oyama, Toshihiko Yamashita, and Mineko Fujimiya
*Corresponding author

Supplementary Results

Bone volume and histological findings were abnormal in OVX rats

An osteoporosis model was constructed using OVX rats and analysed at 4 and 8 weeks after surgery. Micro-computed tomography (micro-CT) showed that the number and volume of trabeculae were obviously reduced in the proximal tibia of OVX rats compared with Sham rats (Supplementary Fig. S1a). Trabecular bone mass was markedly reduced with time after OVX. In quantitative analysis of micro-CT images, bone volume fraction and trabecular number were significantly decreased, while trabecular separation was significantly increased in OVX rats 4 and 8 weeks after OVX (P = 0.008 at 4 weeks, P = 0.002 at 8 weeks, Supplementary Fig. S1b; P = 0.016 at 4 weeks, P = 0.011 at 8 weeks, Supplementary Fig. S1d; P = 0.014 at 4 weeks, P < 0.001 at 8 weeks, Supplementary Fig. S1e). Trabecular thickness was not significantly decreased in OVX rats compared with Sham rats 4 and 8 weeks after OVX (P = 0.6 at 4 weeks, P = 0.274 at 8 weeks, Supplementary Fig. S1c).

Immunophenotype of BM-MSC surface antigens

The immunophenotype of cell surface antigens was similar between OVX-MSCs and Sham-MSCs (Supplementary Fig. S2a). It was unchanged in OVX-MSCs-WJ(+) compared with OVX-MSCs-WJ(-) (Supplementary Fig. S2a).

Abnormal morphology of BM-MSCs derived from OVX rats

Morphological findings of BM-MSCs isolated from OVX rats (OVX-MSCs) were abnormal in phase contrast observations, with short and dull cell protrusions, enlarged cell area, flattened shape, and disordered orientation of cells compared with BM-MSCs isolated from Sham rats (Sham-MSCs). These findings were similarly observed from passage 0 (P0) to P2 (Supplementary Fig. S2b).

Differentiation potential into multiple mesenchymal lineages was altered in OVX-MSCs and OVX-MSCs-WJ(+)

Osteogenic differentiation ability was decreased in OVX-MSCs-WJ(-) compared with Sham-MSCs, as indicated by a decreased number of alkaline phosphatase-positive cells (Supplementary Fig. S2c, upper panels) and alizarin red-positive cells (Supplementary Fig. S2c, middle panels). Conversely, adipogenic differentiation ability was enhanced in OVX-MSCs-WJ(-) compared with Sham-MSCs, as indicated by an increased number of Oil red O-positive lipid droplets in the cytoplasm of BM-MSCs (Supplementary Fig. S2c, lower panels). Osteogenic differentiation was slightly suppressed in OVX-MSCs-WJ(+) compared with

OVX-MSCs-WJ(-), as indicated by a decreased number of alkaline phosphatase-positive cells (Supplementary Fig. S2c, upper panels) and alizarin red-positive cells (Supplementary Fig. S2c, middle panels). Conversely, adipogenic differentiation ability was unchanged in OVX-MSCs-WJ(+) compared with OVX-MSCs, as indicated by the number of Oil red O-positive lipid droplets in the cytoplasm of BM-MSCs (Supplementary Fig. S2c, lower panels).

Distribution of Sham-MSCs, OVX-MSCs-WJ(-), and OVX-MSCs-WJ(+) in OVX rats

PKH26-labelled BM-MSCs were distributed in the bone marrow of the epiphysis in OVX rats (Supplementary Fig. S4a). The number of distributed cells in bone was larger in OVX-Sham-MSCs and OVX-OVX-MSCs-WJ(+) rats compared with OVX-OVX-MSCs-WJ(-) rats on days 1, 3, and 7 after each cell administration (Supplementary Fig. S4b). PKH26 fluorescence-labelled BM-MSCs were also distributed in the lung on days 1, 3, and 7 after each cell administration, but there was no difference between the cell types examined (Supplementary Fig. S4c,d).

Differentiation and maturation of RAW264.7 cell-derived osteoclasts *in vitro*

The macrophage cell line RAW264.7 was differentiated into macrophage-derived osteoclasts by adding receptor activator of nuclear factor κ-B ligand (RANKL) alone or in combination with the MEK inhibitor PD98059 *in vitro* (Supplementary Fig. S5a). Macrophages were fused into multinucleated osteoclasts. Morphologically, osteoclasts became larger and more mature by the addition of RANKL and PD98059 in combination, but not with RANKL alone (Supplementary Fig. S5b). TRACP activity in the culture supernatant of macrophage-derived osteoclasts was increased 72 h after induction with RANKL ($P < 0.05$, Supplementary Fig. S5c).

Differentiation and maturation of mouse BMC-derived osteoclasts *in vitro*

Mouse BMCs were differentiated into macrophage-derived osteoclasts by the addition of RANKL alone or in combination with PD98059 *in vitro* (Supplementary Fig. S6a). Macrophages in the BMC cultures were fused into multinucleated osteoclasts. Morphologically, osteoclasts became larger and more mature with the addition of RANKL and PD98059 in combination, but not with RANKL alone (Supplementary Fig. S6b). TRACP activity in the culture supernatant of macrophage-derived osteoclasts was increased 10 days after induction with RANKL ($P < 0.05$, Supplementary Fig. S6c).

Supplementary Methods

Isolation, culture, and characterisation of BM-MSCs

Bone marrow was collected from Sham and OVX rats. BM-MSCs were harvested by adherent cultures of BMCs as previously described[1]. Briefly, BMCs were harvested from femurs and tibias by flushing whole bone marrow with complete α-modified Eagle's medium (α-MEM; GIBCO BRL, Palo Alto, CA, USA) containing 15% foetal bovine serum (FBS) and 1% penicillin/streptomycin (P/S). Bone marrow cells were suspended as single cells and plated. Cells were grown in complete α-MEM at 37°C and 5% CO_2. Adherent cells grown to confluency were defined as P0. Cells at P2–4 were used for experiments. Surface antigens of BM-MSCs were detected by fluorescence-activated cell sorting (Calibur; BD Bioscience, Franklin Lakes, NJ, USA) using rat surface antigen-specific antibodies to CD90, CD44, CD31, HLA-DR, CD45, CD11b, and CD34. Primary and secondary antibodies used for fluorescence-activated cell sorting are listed in Supplementary Tables S1 and S2.

Quantitative real-time reverse transcription polymerase chain reaction (RT-PCR) of BM-MSCs

Total RNA was extracted using TRI Reagent (Molecular Research Center, Inc.,

Cincinnati, OH, USA), and 1 μg total RNA was reverse-transcribed into cDNA with oligo-dT primers (Promega, Madison, WI, USA) using an Omniscript RT Kit (QIAGEN, Hilden, Germany). Quantitative PCR was performed using an ABI PRISM 7500 Real-Time PCR System (Applied Biosystems, Foster City, CA, USA) with Universal SYBR PCR Master Mix (PerkinElmer, Covina, CA, USA). Thermal cycling conditions were as follows: 40 cycles of a two-step amplification (95°C for 15 s and 60°C for 1 min). Data were analysed using the comparative Ct method (ΔΔCT method). Specific primers used for rat *Opg* are shown in Supplementary Table S5. Rat Gapdh primers acted as an internal standard for RNA integrity and quantity. All PCRs were performed at least in duplicate.

Osteogenic differentiation of BM-MSCs

The *in vitro* differentiation potential of BM-MSCs was confirmed by previously described methods[2]. The multilineage differentiation potential of BM-MSCs was identified by culturing cells. MSCs were plated at a concentration of 4.2×10^3 cells/cm^2 on 4-well slides with MSC culture medium and incubated at 37°C and 5% CO_2. When cells were at 70% confluency 24–48 h later, the medium was replaced with 500 μL of osteogenic differentiation medium. The osteogenic differentiation medium

was replaced every 3 days. After BM-MSCs were cultured in osteogenic differentiation medium for 21 days, they were stained with alkaline phosphatase and alizarin red.

Adipogenic differentiation of BM-MSCs

The multilineage differentiation potential of BM-MSCs was identified by culturing cells. BM-MSCs were plated at a concentration of 2.1×10^4 cells/cm^2 on a 4-well slide with MSC culture medium and incubated at 37°C and 5% CO_2. When the cells were 100% confluent 24–48 h later, the medium was replaced with 500 μL of adipogenic differentiation medium. The adipogenic differentiation medium was replaced every 3 days. After BM-MSCs were cultured in adipogenic differentiation medium for 14 days, they were stained with Oil Red O.

Immunofluorescence staining of BM-MSCs

Immunofluorescence staining of TGF-β1 in BM-MSCs was performed. Each BM-MSC type [Sham-MSCs, OVX-MSCs-WJ(-), and OVX-MSCs-WJ(+); n = 4 per group] was plated in an 8-well chamber slide and cultured for 72 h. WJS (of 0.25 mg/ml) was added to culture medium for 48 h prior to the fixation of cells. Following incubation, BM-MSCs were fixed in 4% paraformaldehyde for 20 min.

Immunofluorescence staining of TGF-β1 was performed by incubating BM-MSCs with primary and secondary antibodies (Supplementary Tables S3 and S4). Nuclei were stained with DAPI (Dojindo Laboratories). Stained sections were observed under a confocal laser-scanning microscope.

Scratch assay

The mobilisation ability of BM-MSCs was evaluated by measuring the area of an open wound at 6 h and 12 h after scratching the cell monolayer. After plating 5×10^4 BM-MSCs [Sham-MSCs, OVX-MSCs-WJ(-), and OVX-MSCs-WJ(+); n = 4 per group] in 35-mm dishes and culturing for 72 h, WJS was added (concentration of 0.25 mg/ml) to culture medium of OVX-MSCs-WJ(+) for 48 h prior to commencing the scratch assay. Crossed straight lines were added to the cell monolayer to create a "scratch" with a P25 pipette tip, and debris was removed by extensive washing. Medium was replaced with 1.5 mL of complete medium. Phase contrast images were taken immediately after making scratches and wound closure was monitored for 12 h. Each experiment was repeated at six times. Open area of the cell monolayer was measured using ImageJ software[3].

Chemotaxis assay

The migration assay was carried out using 12-well culture plates with cell culture inserts containing membranes with 8-μm pores. Upper chambers were loaded with 5 × 10^4 of PKH-26–labelled BM-MSCs with 200 μl of α-MEM containing 15% FBS and 1% P/S, while lower chambers were loaded with 1000 μl of α-MEM containing 15% FBS and 1% P/S. After 24 hours, the upper chamber medium was changed to 200 μl of α-MEM containing 0.5% FBS, and the lower chamber was changed to 1000 μl of α-MEM containing 0.5% FBS with or without 100 ng/ml of SDF-1 (Peprotech, Inc., Rocky Hill, NJ, USA), 100 ng/ml of IL-1β (BioLegend, Inc. San Diego, USA), or 100 ng/ml of IL-6 (BioLegend). Following incubation for 24 h, cells remaining in the upper chamber were removed with cotton swabs, and membranes and lower chamber cells were fixed in 4% paraformaldehyde for 20 min. Cells that migrated to the opposite side of the membrane were stained with DAPI. The number of migrated cells per unit area was determined by counting three randomly selected fields of the membrane with a confocal microscope, and the bottom of the cell culture plate with light a microscope. Recombinant chemokines used for chemotaxis assay were listed in Supplementary Table S6.

Distribution of donor BM-MSCs

Sham-MSCs, OVX-MSCs, and OVX-MSCs-WJ(+) were labelled with a PKH26 Red Fluorescent Cell Linker Kit (Sigma-Aldrich) and administered to OVX rats by tail vein injection 4 weeks after OVX. Rats were euthanised 1, 3, or 7 days after BM-MSC injection, and tibias, lung, liver, and spleen were collected. Bone tissues were immersed in 4% paraformaldehyde for 2 days, and decalcified with 0.5 M ethylenediaminetetraacetic acid (Wako, Osaka, Japan) for 30 days. Lung, liver, and spleen were fixed in 4% paraformaldehyde for 2 days. Frozen sections were stained with DAPI (Dojindo Laboratories, Kumamoto, Japan) at 0.1 mg/mL. The distribution of BM-MSCs expressing red fluorescence in each tissue was observed by confocal laser-scanning microscopy (LSM 510; Carl Zeiss, Oberkochen, Germany). The number of distributed to bone per unit area was determined by counting three randomly selected fields of the view with a confocal microscope in 200 times magnification. The number of distributed to lung per unit area was determined by counting five randomly selected fields of the view with a confocal microscope in 100 times magnification.

Differentiation and maturation of RAW264.7 cell-derived osteoclasts *in vitro*

Osteoclastogenesis of monocytes/macrophages was conducted by modifying

the method of Yonezawa et al.[1]. The murine RAW264.7 monocyte/macrophage cell line (ATCC, Manassas, VA, USA) was used as osteoclast precursors. Cells were grown to subconfluence in a T75 standard flask with Dulbecco's Modified Eagle's Medium (DMEM; Wako Pure Chemical, Osaka, Japan) supplemented with 10% heat-inactivated FBS (Invitrogen, Frederick, MD, USA) and 1% penicillin-streptomycin-glutamine 100× (1% P/S) at 37°C in a humidified atmosphere of 5% CO_2. Subsequently, RAW264.7 cells were transferred to 12-well culture plates and cultured in DMEM supplemented with 15% heat-inactivated FBS and 1% PS at 37°C in a humidified atmosphere of 95% air and 5% CO_2. After 24 h, the culture medium was changed to α-MEM (Wako Pure Chemical) supplemented with 15% heat-inactivated FBS and 1% P/S. For differentiation into mature osteoclasts, RAW264.7 cells (5.0×10^4 cells/well in 12-well culture plates) were cultured for 72 h in the presence of RANKL (100 ng/mL) and/or PD98059 (20 mM), a MAPK inhibitor that accelerates osteoclastogenesis. Cell morphology and supernatant TRACP levels were evaluated.

Differentiation and maturation of mouse BMC-derived osteoclasts *in vitro*

Osteoclastogenesis of mouse BMCs was conducted by modifying the method of Yonezawa et al.[4]. Briefly, mouse BMCs from 7-week-old C57BL/6 mice were used

as osteoclast precursors. Cells were grown to subconfluence in a T25 standard flask with α-MEM supplemented with 10% FBS, 1% P/S, and 100 ng/mL recombinant mouse M-CSF at 37°C in a humidified atmosphere of 5% CO_2. Subsequently, mouse BMCs were transferred to 12-well culture plates and cultured with α-MEM supplemented with 10% FBS, 1% P/S, and 100 ng/mL M-CSF at 37°C in a humidified atmosphere of 95% air and 5% CO_2. After 48 h, the culture medium was changed to α-MEM supplemented with 15% FBS, 1% P/S, and 100 mg/mL M-CSF. For differentiation into mature osteoclasts, mouse BMCs (6.0×10^5 cells/well in 12-well culture plates) were cultured for 10 days in the presence of RANKL (100 ng/mL) with or without PD98059 (20 mM). Cell morphology and supernatant TRACP levels were evaluated.

Measurement of TRACP levels in culture supernatant of osteoclasts

Culture supernatants of osteoclasts were collected and stored at -80°C until use. TRACP levels in culture supernatants were measured with a TRACP & ALP Assay Kit (Takara Bio, Inc., Shiga, Japan) according to the manufacturer's instructions.

Supplementary References

1 Javazon, E. H., Colter, D. C., Schwarz, E. J. & Prockop, D. J. Rat marrow stromal cells are more sensitive to plating density and expand more rapidly from single-cell-derived colonies than human marrow stromal cells. *Stem Cells* **19**, 219-225, doi:10.1634/stemcells.19-3-219 (2001).

2 Romanov, Y. A., Svintsitskaya, V. A. & Smirnov, V. N. Searching for alternative sources of postnatal human mesenchymal stem cells: candidate MSC-like cells from umbilical cord. *Stem Cells* **21**, 105-110, doi:10.1634/stemcells.21-1-105 (2003).

3 Schneider, C. A., Rasband, W. S. & Eliceiri, K. W. NIH Image to ImageJ: 25 years of image analysis. *Nat Methods* **9**, 671-675 (2012).

4 Yonezawa, T. *et al.* Biselyngbyaside, isolated from marine cyanobacteria, inhibits osteoclastogenesis and induces apoptosis in mature osteoclasts. *Journal of cellular biochemistry* **113**, 440-448, doi:10.1002/jcb.23213 (2012).

Supplementary Figure Legends

Supplementary Figure S1. Abnormalities of bone tissues in OVX rats.

(a) Representative micro-CT images of tibias at 4 and 8 weeks after Sham or OVX operation in rats. (b–e) Quantitative changes in trabecular parameters, including

trabecular bone volume expressed as b: percentage of total tissue volume (BV/TV), c: trabecular thickness (Tb.Th), d: trabecular number (Tb.N), and e: trabecular separation (Tb.Sp). *$P < 0.05$. Data are expressed as mean ± SE of 4–5 animals.

Supplementary Figure S2. Characterisation of Sham-MSCs, OVX-MSCs-WJ(-), and OVX-MSCs-WJ(+).

(a) Immunophenotype expression of cell surface antigens analysed by flow cytometry in Sham-MSCs (upper panels), OVX-MSCs-WJ(-) (middle panels), and OVX-MSCs-WJ(+) (lower panels). (b) Phase contrast observations of Sham-MSCs (left panel) and OVX-MSCs (right panel). Images were obtained from P0, P1, and P2 cells at 12 weeks after surgery. Bar: 100 μm. (c) Osteogenic and adipogenic differentiation of Sham-MSCs (left panel), OVX-MSCs-WJ(-) (middle panel), and OVX-MSCs-WJ(+) (right panel). Images were obtained at 14 days after culture with osteogenic or adipogenic differentiation medium. Bone matrices are stained blue using an alkaline phosphatase (ALP) staining kit, and red by alizarin red staining kit. Fat droplets are stained red with Oil red O staining. Bar: 100 μm.

Supplementary Figure S3. WJS enhanced the mobilisation of OVX-MSCs.

(a) Chemotaxis assay. Phase contrast observations of BMCs mobilised into the bottom of the lower chamber without any chemokines (upper panel), with SDF-1 (middle upper panel), IL-1β (middle lower panel), or IL-6 (lower panel). Images were obtained 24 h after culture with SDF-1, IL-1β, or IL-6. Bar: 500 μm. SDF-1, stromal cell-derived factor 1; IL-1β, interleukin-1 beta; IL-6, interleukin-6. (b) Numbers of migrated BM-MSCs in the bottom of lower chambers counted under a light microscope. Values represent mean ± SE of Sham-MSCs, OVX-MSCs-WJ(-), and OVX-MSCs-WJ(+). *$P <$ 0.05. Data are expressed as mean ± SE of three BM-MSC cultures.

Supplementary Figure S4. Distribution of Sham-MSCs, OVX-MSCs, and OVX-MSCs-WJ(+) in OVX rats.

Distribution of administered Sham-MSCs, OVX-MSCs, and OVX-MSCs-WJ(+) in OVX rats at days 1, 3, and 7. DAPI was used for counterstaining nuclei (blue). White dotted line indicated the edge of trabeculae bone. (a) BM-MSCs were detected in bone using the immunofluorescence marker PKH26 (red). Bar: 50 μm. (b) Numbers of BM-MSCs detected in bone tissues. *$P < 0.05$. Values represent mean ± SE of Sham-MSCs rats, OVX-OVX-MSCs-WJ(-) rats, OVX-OVX-MSCs-WJ(+) rats, n = 4 in day1 and day3 per group and n = 3 in day 7 per group. (c) BM-MSCs were detected in

the lung. Bar: 100 µm. (d) Numbers of detected BM-MSCs counted under a light microscope. Values represent mean ± SE of Sham-MSCs, OVX-MSCs-WJ(-), and OVX-MSCs-WJ(+). *$P < 0.05$. Data are expressed as mean ± SE of three BM-MSC cultures.

Supplementary Figure S5. Differentiation of RAW264.7 cells into osteoclasts.

(a) Experimental protocol to induce macrophage-derived osteoclasts using RAW264.7 cells. (b) Phase contrast observations of RAW264.7 cells cultured without RANKL (left panel), with RANKL (middle panel), and with RANKL and PD98059 (right panel). Images were obtained 72 h after adding RANKL with or without PD98059 to the culture medium. Bar: 500 µm in upper panel, 100 µm in lower panel. (c) TRACP levels in the supernatant of RAW264.7 cell-derived osteoclasts. *$P < 0.05$. Data are expressed as mean ± SE of three experiments.

Supplementary Figure S6. Differentiation of mouse BMCs into osteoclasts.

(a) Experimental protocol to induce macrophage-derived osteoclasts using BMCs from mice. (b) Phase contrast observations of BMCs cultured without RANKL (left panel), with RANKL (middle panel), and with RANKL and PD98059 (right panel). Images

were obtained 10 days after adding RANKL with or without PD98059 to the culture medium. Bar: 500 μm in upper panel, 100 μm in lower panel. (c) TRACP levels in the supernatant of BMC-derived osteoclasts. *$P < 0.05$. Data are expressed as mean ± SE of six experiments.

Supplementary Table Legends and Captions

Supplementary Table S1. Primary antibodies used for fluorescence-activated cell sorting analysis

Ms, mouse; Rt, rat; Hu, human.

Supplementary Table S2. Secondary antibodies used for fluorescence-activated cell sorting analysis.

Dnk, donkey.

Supplementary Table S3. Primary antibodies used for immunofluorescence.

RANK, receptor activator of NF-κB; TRACP, tartrate-resistant acid phosphatase; IL-1β, interleukin-1 beta; IL-6, interleukin-6; TGF-β, transforming growth factor β1; Ms, mouse; Rt, rat; Hu, human.

Supplementary Table S4. Secondary antibodies used for immunofluorescence.

Dnk, donkey.

Supplementary Table S5. Primer sequences used for quantitative RT-PCR of rat BM-MSCs.

Opg, osteoprotegerin; *Gapdh*, glyceraldehyde 3-phosphate dehydrogenase.

Supplementary Table S6. Recombinant chemokines used for chemotaxis assay.

SDF-1, stromal-cell derived factor-1; IL-1β, interleukin-1 beta; IL-6, interleukin-6; Rt, rat.

Supplementary Table S7. Primer sequences used for quantitative RT-PCR of RAW264.7 cell-derived osteoclasts and mouse bone marrow-derived osteoclasts.

Nfatc1, nuclear factor of activated T cells; *Cath-k*, cathepsin K; *Clc7*, chloride channel 7; *Atp6i,* ATPase, H^+ transporting, (vacuolar proton pump) member I; *Dc-stamp*, dendritic cell-specific transmembrane protein; *Gapdh*, glyceraldehyde 3-phosphate dehydrogenase.

Supplementary Tables

Supplementary Table S1. Primary antibodies used for fluorescence-activated cell sorting analysis

Antibody	Species	Reactivity	Manufacture

**Immunophenotype**			
CD90(Thy1.1)	Ms-IgG1	Ms, Rt	BioLegend
CD44	Ms-IgG1	Rt	BioLegend
CD31	Ms-IgG1	Rt	AbD Serotec
HLA-DR	Ms-IgG1	Rt	BioLegend
CD45	Ms-IgG2a	Rt	BioLegend
CD11b	Ms-IgG2a	Rt	BioLegend
CD34	Rb	Ms, Rt, Hu	BioLegend
Mouse IgG1		-	BD Bioscience
Mouse IgG2a		-	BD Bioscience
Rabbit IgG		-	BioLegend

Supplementary Table S2. Secondary antibodies used for fluorescence-activated cell sorting analysis

Antibody	Species	Conjugate	Manufacture
**Immunophenotype**			
Anti-mouse IgG	Dnk	FITC	Chemicon
Anti-rabbit IgG	Dnk	FITC	Chemicon

Supplementary Table S3. Primary antibodies used for immunofluorescence

Antibody	Species	Reactivity	Manufacturer
**Immunofluorescence**			
RANK	Rt	Ms, Rt, Hu	Santa Cruz Biotechnology
TRACP	Ms	Ms, Rt, Hu	BioLegend
IL-1β	American Hamster	Ms, Rt	BioLegend
IL-6	Goat	Ms, Rt	Santa Cruz Biotechnology
TGF-β1	Rb	Ms, Rt, Hu, Pig	Abcam

Supplementary Table S4. Secondary antibodies used for immunofluorescence

Antibody	Species	Conjugate	Manufacturer
**Immunofluorescence**			
Anti-mouse IgG	Dnk	Cy3	Chemicon
Anti-rabbit IgG	Dnk	Cy3	Jackson Laboratory
Anti-American Hamster IgG	Goat	DyLight649	BioLegend
Anti-Goat IgG	Dnk	Cy3	Merck Millipore

Supplementary Table S5. Primer sequences used for quantitative PCR of BM-MSCs

Gene	Locus	Direction	Sequence
Opg	NM_012870	forward	5’- *gccaacactgatggagcagat* -3’
		reverse	5’- *tcttcattcccaccaactgatg* -3’
Gapdh	NM_017008	forward	5’- *caaggatactgagagcaagaga* -3’
		reverse	5’- *aggcccctcctgttgttat* -3’

Supplementary Table S6. Recombinant chemokines used for chemotaxis assay

**Recombinant**	**Reactivity**	**Manufacturer**
SDF-1α	Rt	Peprotech
IL-1β	Rt	BioLegend
IL-6	Rt	BioLegend

Supplementary Table S7. Primer sequences used for quantitative RT-PCR of RAW264.7 cell-derived osteoclasts and mouse bone marrow-derived osteoclasts

Gene	Locus	Direction	Sequence
Nfatc1	NM_ 1164111	forward	5’- *cagtgtgaccgaagatacctgg*-3’
		reverse	5’- *tcgagacttgatagggacccc*-3’

Cath-k	NM_007802	forward	5’- aatacctccctctcgatcctaca-3’
		reverse	5’- tggttcttgactggagtaacgta-3’
Clc7	NM_011930	forward	5’- gacaacagcgagaatcagctc-3’
		reverse	5’- ccaatgagggcacagataacc-3’
Atp6i	NM_ 001167784.	forward	5’- *attgccagctttcgggagac*-3’
		reverse	5’- *cggatcttctgtccgatctgc*-3’
Dc-stamp	NM_001289506	forward	5’- ctgtgtcctcccgctgaataa-3’
		reverse	5’- agccgatacagcagatagtcc-3’
Gapdh	NM_01289726	forward	5’- tggccttccgtgttcctac-3’
		reverse	5’- gagttgctgttgaagtcgca-3’

Supplementary Figure S1

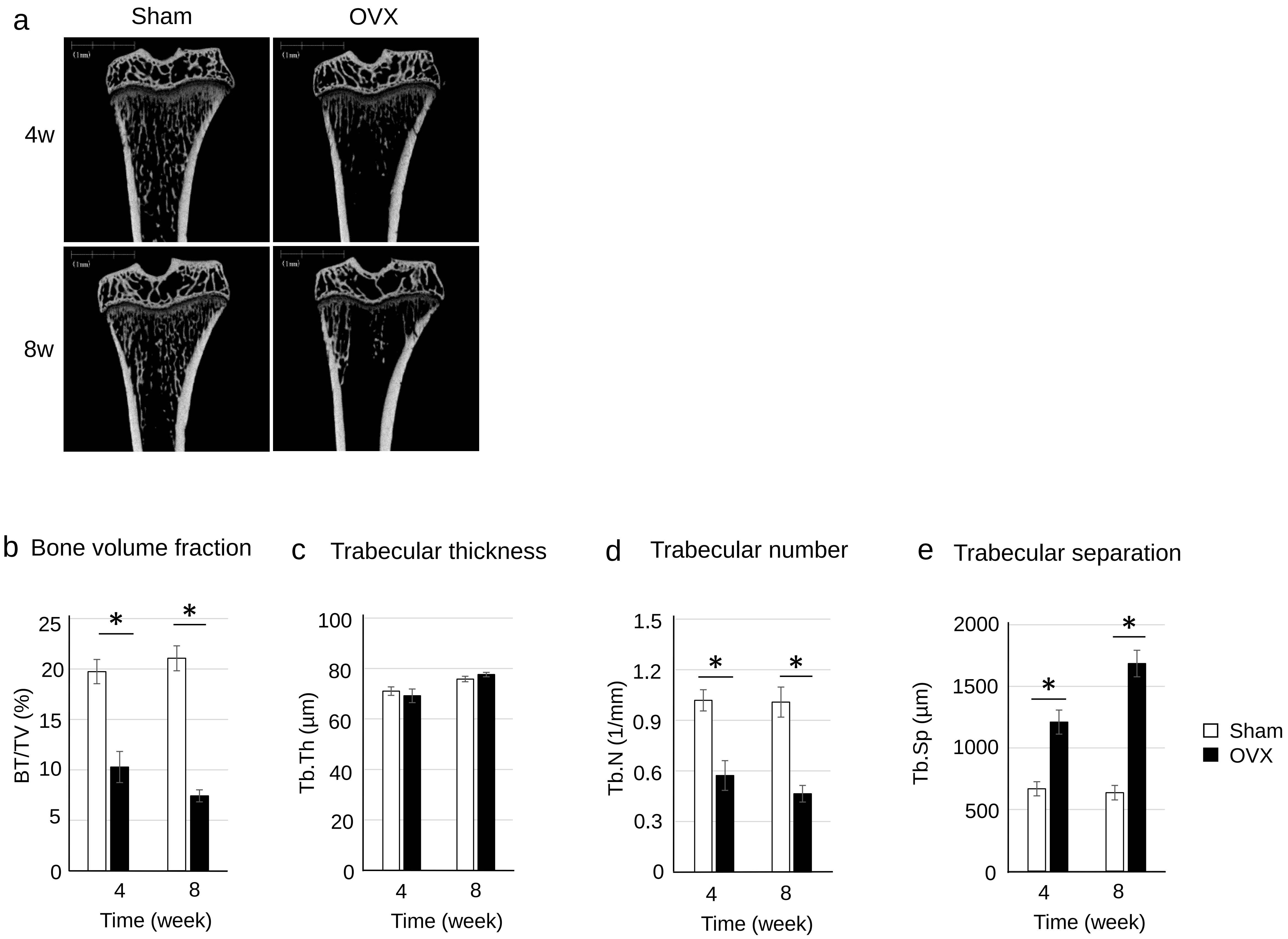

Supplementary Figure S2

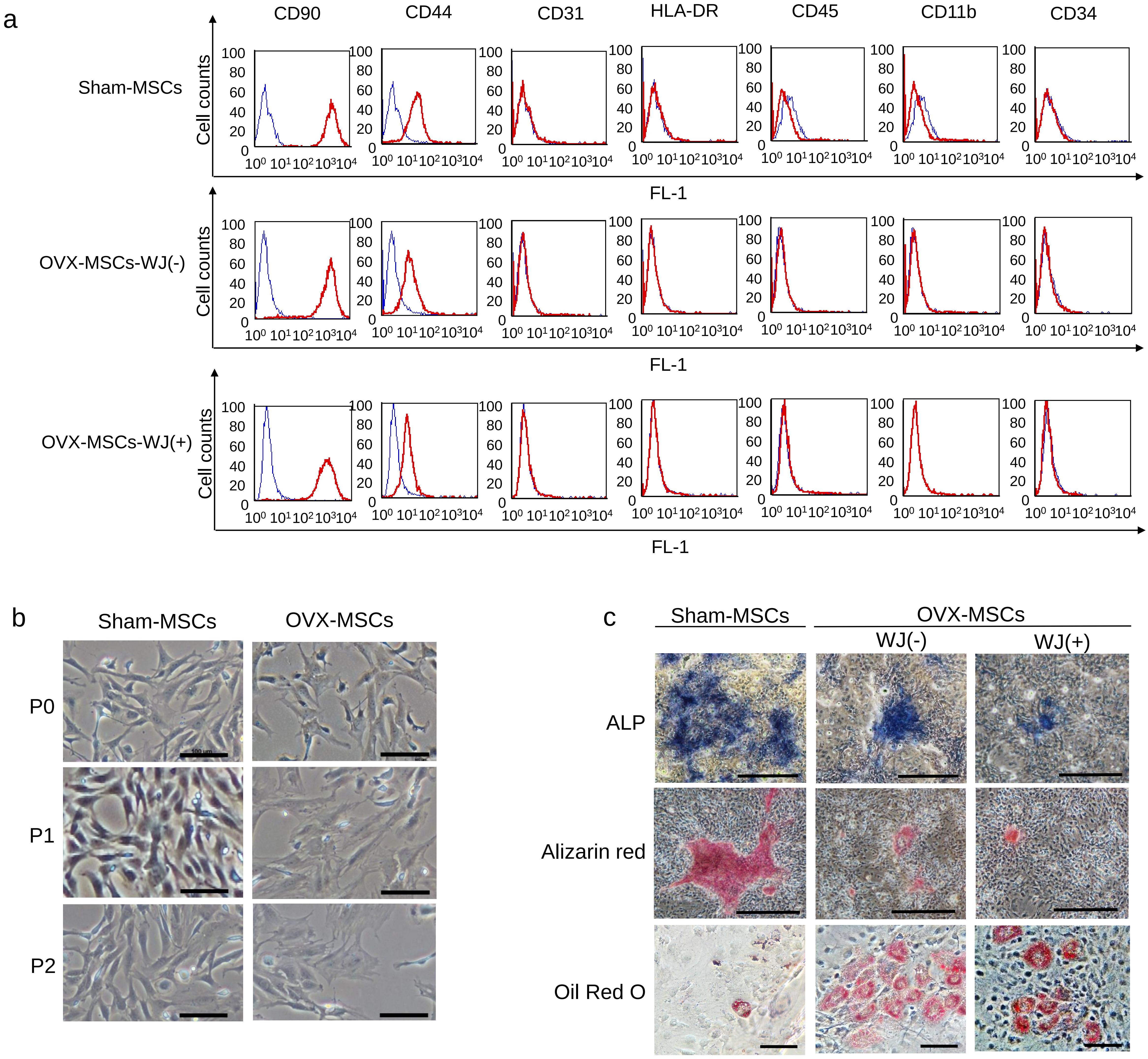

Supplementary Figure S3

a

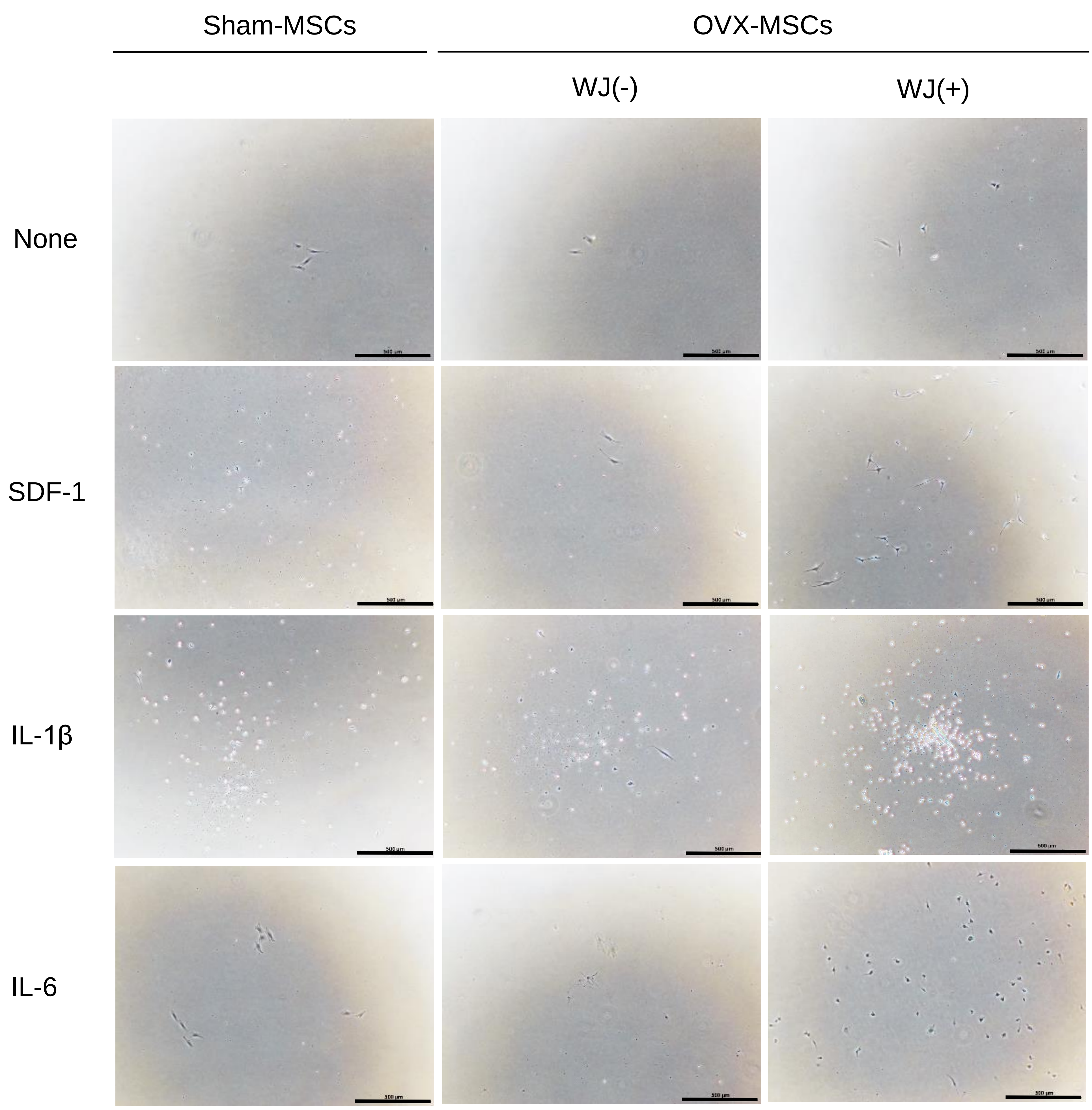

b

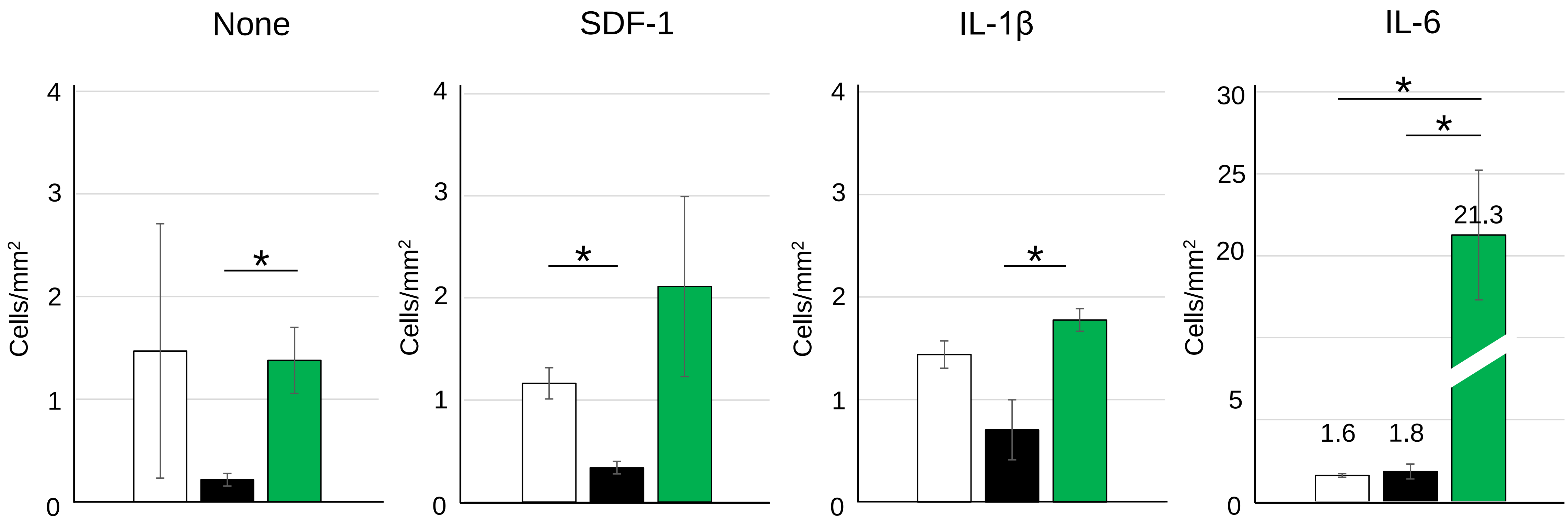

Supplementary Figure S4

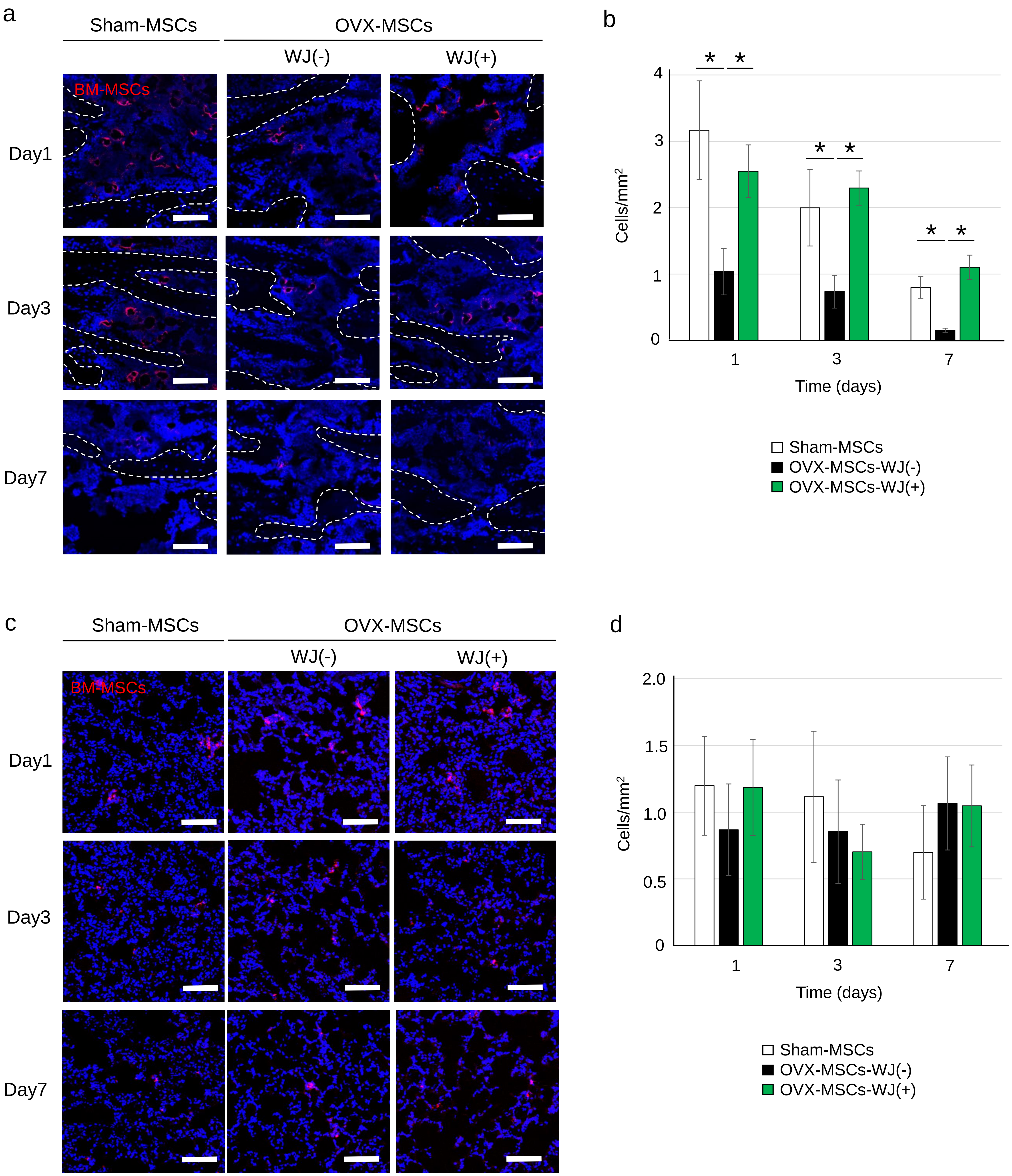

Supplementary Figure S5

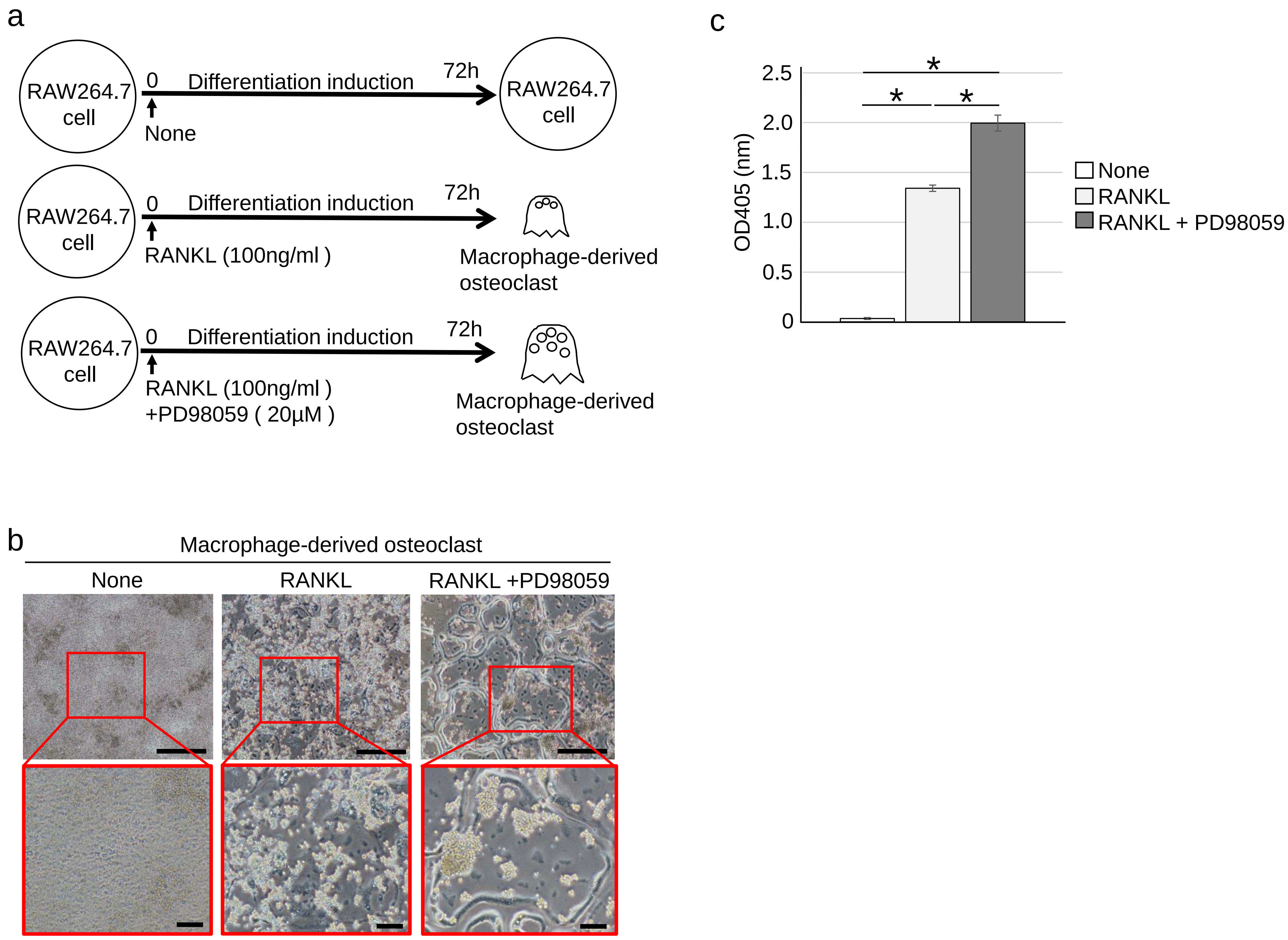

Supplementary Figure S6

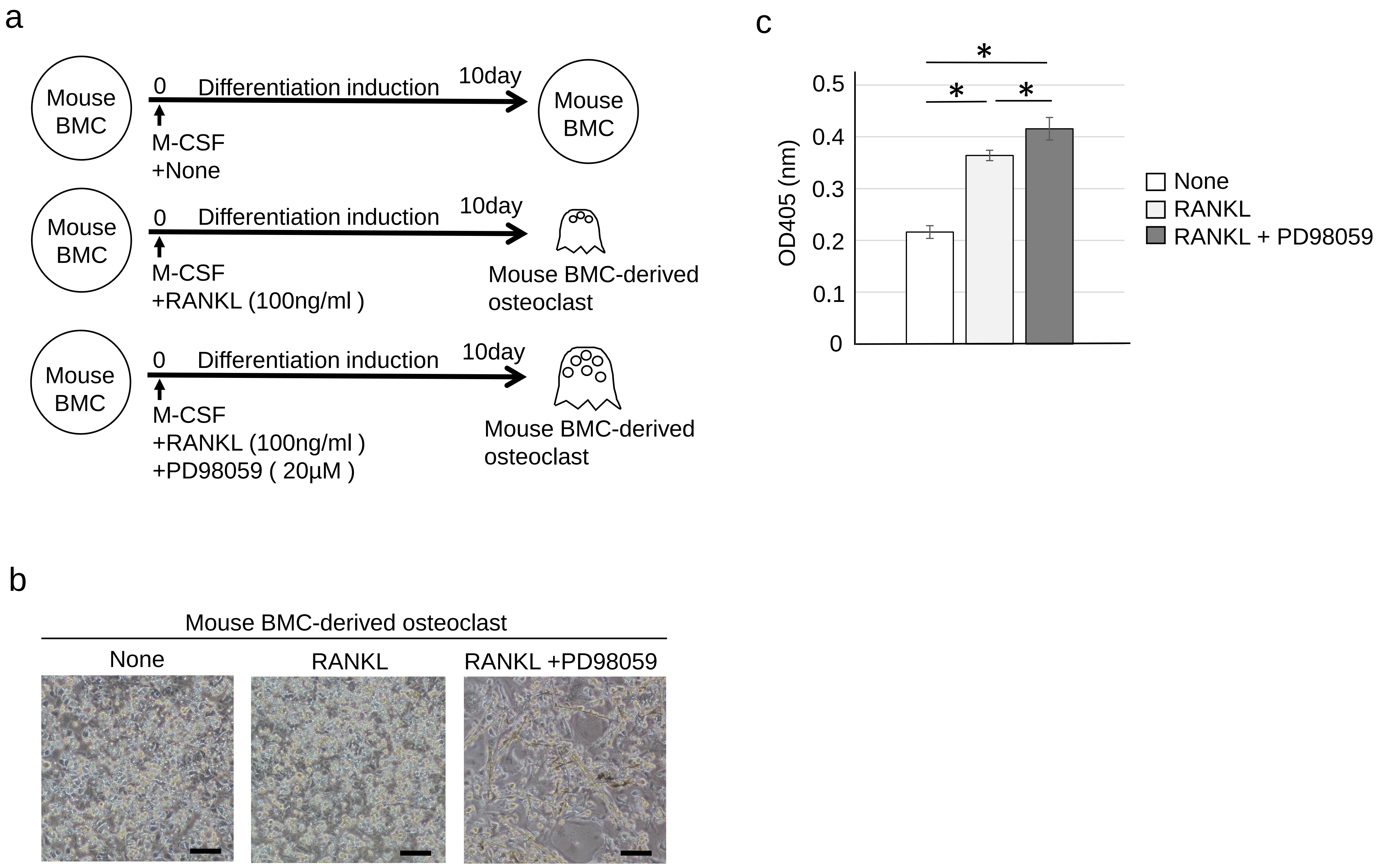

Supplement: Supplementary file 1 — Supplementary information [file 41598_2018_19516_MOESM1_ESM.pdf]
